# Physiological activation regimes reveal distinct autonomic mechanisms underlying operational performance under cognitive workload and stress

**DOI:** 10.3389/fnrgo.2026.1946611

**Published:** 2026-10-02

**Authors:** Ludovica Di Pompeo, Michele Tritto, Francesco Romano, David Perpetuni, Daniela Cardone, Arcangelo Merla

**Affiliations:** 1 BioEngineering Lab, Department of Engineering and Geology, University of Chieti-Pescara “Gabriele D'Annunzio,” Pescara, Italy; 2 Next2u, Pescara, Italy

**Keywords:** cognitive workload, heart rate variability (HRV), mental stress, operational performance, physiological activation

## Abstract

Human performance in safety-critical environments depends on the interaction between mental workload, stress, autonomic regulation, and behavior. This study investigated whether associations between heart rate variability (HRV) and performance varied across physiological activation ranges and operational contexts during a multi-task flight simulation implemented through the AeroStim platform. Forty-two healthy participants (25 ± 5 years) performed either a Workload-Only condition (WO; *n* = 20), comprising Tracking and System Monitoring tasks, or a Stress-Oriented condition (SO; *n* = 22), in which additional stress-inducing tasks were concurrently administered. ECG was continuously recorded using an EqVital wearable T-shirt. Linear and non-linear HRV indices were extracted and normalized within participants using Min-Max scaling. Gaussian Mixture Modeling of normalized SDNN identified three relative, subject-specific activation ranges (*Easy, Medium*, and *Hard*), with classification showing high stability across alternative normalization procedures. HRV-performance associations were assessed using Spearman correlations with permutation-based inference and Benjamini–Hochberg correction, while performance differences were evaluated using non-parametric tests. HRV-performance correlations varied across activation ranges and contexts; none passed FDR correction, so these should be considered exploratory. In WO, associations involved LF in the Easy range, SDNN, SD2, and LF in the Medium range, and SDNN, LF/HF, and Sample Entropy in the Hard range. In SO, associations in the Medium range extended to vagal, global variability, and non-linear complexity indices, while Hard-range associations mainly involved vagal and LF-related measures. Omission rate was lower at Medium than Easy activation in WO, providing partial support for an intermediate activation advantage, whereas no consistent performance optimum emerged across contexts. Between-condition comparisons revealed a robust speed-accuracy trade-off, with WO producing faster but less accurate responses and SO producing slower but more accurate responses. Sensitivity analysis further indicated that differences in exercise windows affected HRV metrics in a metric-dependent manner, with greater deviations generally observed for frequency-domain measures. The findings suggest that HRV-performance relationships are context- and activation-dependent rather than captured by a single universal marker. The results support the potential of regime-based, multimodal HRV monitoring for adaptive human-machine systems, while highlighting the need for standardized recording durations, independent autonomic markers, and validation across individuals and real operational environments.

## 1 Introduction

In safety-critical or operational scenarios human performance emerges from the dynamic interplay between cognitive workload (MWLD), stress, and physiological regulatory mechanisms. Degradations in this interplay have direct operational consequences, as human factors have been identified as a major contributor to aviation accidents and incidents. A review of more than 200 commercial air transport events estimated that human factors contributed to approximately 75% of the analyzed events (Kharoufah et al., 2018), consistent with Federal Aviation Administration (FAA) estimates indicating that human error is implicated in roughly two-thirds to three-quarters of aviation accidents and incidents (FAA). Characterizing the operator's physiological state in real time is therefore a prerequisite for safety and performance management across safety-critical domains.

MWLD is defined as the ratio between the attentional, memory, and executive resources required to perform a task and those available at a given moment (Wickens, 2008; Heard et al., 2018). Stress, in contrast, refers to an acute psychophysiological response to environmental demands perceived as exceeding or threatening an individual's regulatory capacity (Qiu et al., 2022). Both phenomena can influence autonomic regulation, which contributes to the cardiovascular response through the dynamic interplay between sympathetic and parasympathetic activity. This autonomic-cardiovascular relationship is bidirectional and is also shaped by baroreflex, respiratory and metabolic inputs. Heart rate variability (HRV) provides a non-invasive and practical index of cardiac autonomic regulation and may provide indirect information about changes in the operator workload, stress, readiness and recovery.

HRV encompasses a broad spectrum of metrics that characterize autonomic regulation across multiple analytical domains. However, HRV indices are also influenced by other physiological factors, including respiratory rate, tidal volume, body posture, physical activity, circadian phase, recording duration, signal quality, and signal processing choices. Therefore, HRV indices should be interpreted as context-dependent measures of cardiac variability and autonomic regulation rather than as direct measures of autonomic activity. In the time domain, the standard deviation of NN intervals (SDNN) indexes total autonomic variability, integrating both sympathetic and parasympathetic contributions across a range of timescales (Shaffer et al., 2014; Kleiger et al., 2005); the root mean square of successive differences (RMSSD) reflects short-term vagal modulation and is considered the most robust time-domain marker of parasympathetic activity (Thayer and Lane, 2000; Shaffer and Ginsberg, 2017); the proportion of successive NN interval differences exceeding 50 ms (pNN50) provides a complementary index of high-frequency parasympathetic tone (Mietus et al., 2002). Poincaré plot-derived indices SD1 and SD2 decompose beat-to-beat variability into its short-term (vagally mediated) and long-term (combined sympathovagal) components, respectively, and have been used to monitor autonomic responses to cognitive workload in operational contexts (Brennan et al., 2001; Laborde et al., 2017). In the frequency domain, high-frequency power (HF: 0.15–0.40 Hz) is primarily associated with cardiac vagal modulation and is influenced by respiratory sinus arrhythmia (Berntson et al., 1997); low-frequency power (LF: 0.04–0.15 Hz) reflects a mixture of autonomic and baroreflex-related influences rather than a specific sympathetic component (Billman, 2011; Reyes del Paso et al., 2013); and the LF/HF ratio is commonly used as a descriptive index of the relative distribution of LF and HF power under cognitive demands, although its interpretation as a direct marker of sympathetic dominance remains debated (Malik et al., 1996; Billman, 2011). Beyond linear indices, non-linear complexity measures have gained increasing attention in operational psychophysiology as they capture dynamic properties of the RR series that are not accessible through time- or frequency-domain analyses, and that are differentially sensitive to autonomic dysregulation and cognitive demand (Laborde et al., 2017; Melillo et al., 2011). Sample Entropy (SampEn) quantifies the irregularity and unpredictability of the RR series, with higher values indicating greater signal complexity; decreases in SampEn under acute stress and high cognitive load have been documented in both laboratory and operational settings (Richman and Moorman, 2000; Zhu and Bo, 2019; Castaldo et al., 2015). Fuzzy Entropy (FuzzyEn) extends this approach by replacing the hard binary boundary of SampEn with a continuous similarity function, yielding more stable estimates at shorter recording lengths and with smaller sample sizes, properties particularly advantageous in epoch-based operational monitoring (Chen et al., 2009; Liu et al., 2011). Permutation Entropy (PermEn) captures the ordinal complexity of the RR series based on the rank structure of local amplitude patterns rather than their absolute values, rendering it robust to non-stationarity and amplitude artifacts; higher PermEn reflects greater temporal irregularity in the beat-to-beat pattern, and reductions have been associated with increased sympathetic dominance during sustained cognitive effort (Bandt and Pompe, 2002; Bian et al., 2012). The correlation dimension D2, estimated via the Grassberger–Procaccia algorithm, quantifies the fractal dimensionality of the reconstructed phase-space attractor of the RR series: lower D2 values indicate a more deterministic, low-dimensional cardiac dynamics, and reductions during acute stress have been interpreted as a loss of non-linear complexity consistent with the complexity-loss hypothesis of physiological regulation under load (Lipsitz and Goldberger, 1992; Javorka et al., 2008). Wearable electrocardiography (ECG) and photoplethysmography (PPG) technologies have made ecological acquisition of this multidomain HRV profile increasingly feasible across operational settings (Elfouly and Alouani, 2025).

Multidomain HRV metrics can provide important insights into how autonomic regulation responds to changes in workload and stress. This relationship is supported by two complementary lines of research. The first analyzes autonomic responses to different task demands and stressful conditions by assessing changes in HRV metrics. For example, in studies of pilot mental workload, HRV decreases during high-demand flight phases, such as landing, and increases again during low-demand phases, such as cruise, following the progression of task demands (Yuan et al., 2025). In surgery, non-linear indices such as SampEn and D2 distinguish expert and novice operators engaged in comparable laparoscopic tasks, while acute work-related stress conditions are associated with reductions in RMSSD, HF, and entropy-based measures (Pérez-Salazar et al., 2024; Stephenson et al., 2021). Similarly, in military contexts, continuous HRV monitoring has highlighted dynamic variations in SDNN, RMSSD, LF/HF, and non-linear complexity indicators during physical exertion, sleep deprivation, and psychological stress, sometimes preceding a measurable decline in performance (Burlacu et al., 2026). A second line of research explores the possibility of estimating or classifying load and stress states, based on HRV. In this context, machine learning models integrating linear and non-linear HRV features have shown promising results in identifying pilots' mental workload during real-world flight operations (Wang et al., 2024). Overall, these findings indicate that HRV is not only sensitive to changes in load and stress, but also informative for estimating and, potentially, distinguishing them.

It is crucial to emphasize that stress and workload do not act as independent factors and that their combined effect on performance and autonomic regulation is not simply additive. A moderate stress component can act a constructive interference in workload processing, enhancing attentional selectivity and supporting the allocation of resources required for a demanding task. This effect is consistent with both the inverted U-shaped relationship between arousal and performance (Yerkes and Dodson, 1908) and compensatory control theories of effort regulation under conditions of high workload and stress (Hockey, 1997). However, stress can also produce destructive interference: because stress and workload draw on a shared and limited pool of attentional and executive resources already committed to the primary task. This competition leads to the narrowing of attention described by Attentional Control Theory (Eysenck et al., 2007) and manifests physiologically as an amplified or less regulated autonomic response.

This dual potential is visible in several operational domains. In aviation, moderate acute stress superimposed on high mental workload during simulated flight has been shown to trigger further recruitment of the executive control network, allowing pilots to cope with the combined demands through increased neural effort rather than through a decline in performance: a case of constructive interference, in which stress-related activation supports, rather than impairs, the allocation of task-relevant resources (Causse et al., 2024). This pattern contrasts with what has been observed in other operational domains, where the same combination of stress and workload can be disruptive. In air traffic control, time pressure and communication demands that induce stress arise precisely from the fact that controllers must simultaneously monitor multiple aircrafts—a condition of high workload—and it is precisely under this combined pressure that attention narrows and controllers devote less time to monitoring variables considered of lower priority, sometimes contributing to operational errors (Durso et al., 1998): a case of destructive interference, in which stress aggravates rather than compensates for an already saturated resource pool. In minimally invasive surgery, where the surgeon operates through an instrumental interface with reduced sensory feedback, a dynamic opposite to that of low-load contexts emerges: when high levels of stress overlap with already technically demanding and high-workload phases of the procedure, errors, an increase in operating times, and a deterioration in fine motor skills and gestural precision can occur (Pérez-Salazar et al., 2024). This shows that the effect of stress is not intrinsically positive or negative, but it depends on the level of workload it occurs within. In motorsport, where drivers must process information in fractions of a second, manage vehicle setup, and anticipate opponents' behavior under conditions of high physical risk, reductions in time-domain HRV indices following competitive sessions indicate a shift toward sympathetic dominance and reduced self-regulation, resulting from the combined—and not easily separable—influence of prolonged cognitive demand, emotional stress, and physical load (Marcos-Frutos et al., 2026).

Disentangling constructive from destructive interference first requires the ability to independently induce stress and workload, through controlled stimulation paradigms such as the AeroStim flight simulation platform used in the present study (Di Cesare et al., 2024). Once this experimental control is achieved, it becomes possible to analyze how the two factors interact in determining operational performance and the underlying physiological regulation.

Most existing studies model HRV-performance relationships by comparing predefined task difficulty conditions or by examining global correlations across the entire physiological range. These approaches implicitly assume linearity and homogeneity in the association between physiological activation and behavioral performance, potentially masking qualitatively distinct autonomic regimes in which different HRV indices—linear and non-linear alike—may have differential predictive value. In this context, autonomic regimes can be broadly interpreted as low, medium and high levels of physiological activation, reflecting different balances between parasympathetic and sympathetic activity, from vagal-dominant and low-arousal states to intermediate adaptive engagement and highly sympathetic activation (Shaffer and Ginsberg, 2017; Laborde et al., 2017). Furthermore, it remains unclear whether HRV metrics reflect actual performance deterioration or, more subtly, the compensatory regulatory mechanisms that preserve performance under demand—a distinction with direct implications for adaptive monitoring.

To address this gap, the present study adopts a regime-based framework in which relative physiological activation ranges are derived from the physiological signal. Within-participant Min-Max-normalized SDNN was partitioned into three ranges—*Easy, Medium*, and *Hard*—using a Gaussian mixture model (GMM). The three-range structure was specified a priori, while the GMM provided empirical support for the resulting classification. These ranges represent relative, subject-specific activation states rather than universal physiological thresholds. Two operational contexts were defined: Workload-Only (WO), which cognitive load is manipulated independently; and Stress-Oriented (SO), combining the workload demands of the primary tasks with additional concurrent secondary-task and potential stressor-related demands. In both contexts, participants performed the same primary tasks: Tracking task, which simulates maintaining the aircraft's course, and System Monitoring task requiring active detection of anomalies, both essential for piloting the simulated aircraft. In the WO context, cognitive demands were modulated solely by varying the difficulty of the primary tasks, according to predefined workload levels. In the SO context, the same workload modulation was applied, to which a series of secondary tasks—Communication, Stroop, Spatial Stroop and Resource Management—were added to increase divided attention, interference, and time pressure, thereby creating conditions known to elicit stress responses in operational environments (Staal, 2004).

By applying the same regimen-based framework to both operating contexts, it is possible to investigate the relationship between HRV and performance at different levels of activation when workload acts alone, and whether this relationship changes when additional concurrent tasks intended to elicit stress responses are introduced—and, in both cases, whether some activation ranges are associated with more sustainable performance than other.

Therefore, the specific objectives of this study are: (i) to characterize the associations between HRV and performance outcomes across different levels of physiological activation under conditions involving variations in cognitive workload alone; (ii) to examine how these HRV-performance associations differ in the presence of additional concurrent demands across activation ranges; and (iii) to identify whether distinct activation ranges—among the three defined for each operating context—are associated with different and potentially more sustainable performance profiles. Adaptive psychophysiological monitoring systems require workload models capable of detecting not only performance degradation, but also the physiological cost associated with maintaining performance—a capability that depends on identifying physiologically activation states characterize by distinct HRV-performance patterns (Teo et al., 2020). By providing a physiologically grounded, regime-based characterization of HRV-performance relationships across a comprehensive set of linear and non-linear autonomic indices, this study aims to contribute both to the theoretical understanding of autonomic regulation under operational demands and to the development of adaptive human-machine systems for performance monitoring and sustainable performance management.

## 2 Materials and methods

### 2.1 Participants

The experimental activity was conducted at the BioEngineering Lab of the Gabriele D'Annunzio University, under controlled conditions, on a sample of 50 university students with mean age = 25.2 ± 5.3 years and with no overt history of cardiovascular or neurological disorders. Gender was not recorded for privacy reasons, so potential sex-related differences could not be examined and should be considered when interpreting the findings. Participants were recruited voluntarily and received no financial compensation. All subjects provided written informed consent prior to the start of the experiment. The experimental procedures and operating instructions were provided in the participants 'native language to ensure full understanding of the required activities. The study was approved by the Local Ethical Board (CeTRA #0049263/24). Out of the 50 participants, eight were excluded from the analyses: two because the physiological recording files were corrupted and six because performance data were unavailable due to technical acquisition failures. Exclusion comprised five participants from the WO condition and three from the SO condition, resulting in a final sample of 42 subject.

### 2.2 Workload and stress induction

Desired levels for each operating context (WO, SO) were administered to the subjects via Next2U (Next2U, Via dei Peligni, Pescara, Italy) AeroStim software suite (Figure 1; Di Cesare et al., 2024), developed as an evolution of the Multi-Attribute Task Battery II (MATB-II; Santiago-Espada et al., 2011). AeroStim extends the original MATB-II framework, transforming it from a set of standardized tasks to a more flexible and configurable environment thanks to its modular and extensible architecture: it allows for the customization of tasks and stimuli without modifying the underlying software structure. This modularity allows for the manipulation cognitive load and the introduction of additional stressor-related demands (e.g., time pressure, auditory stress, and task interference). AeroStim also ensures the recording of the logs of all the relevant events (e.g., stimulus onset, responses, errors, task transitions, and presentation of stressors) with high-resolution timestamps that can be time-synchronized with physiological signals.

**Figure 1:**
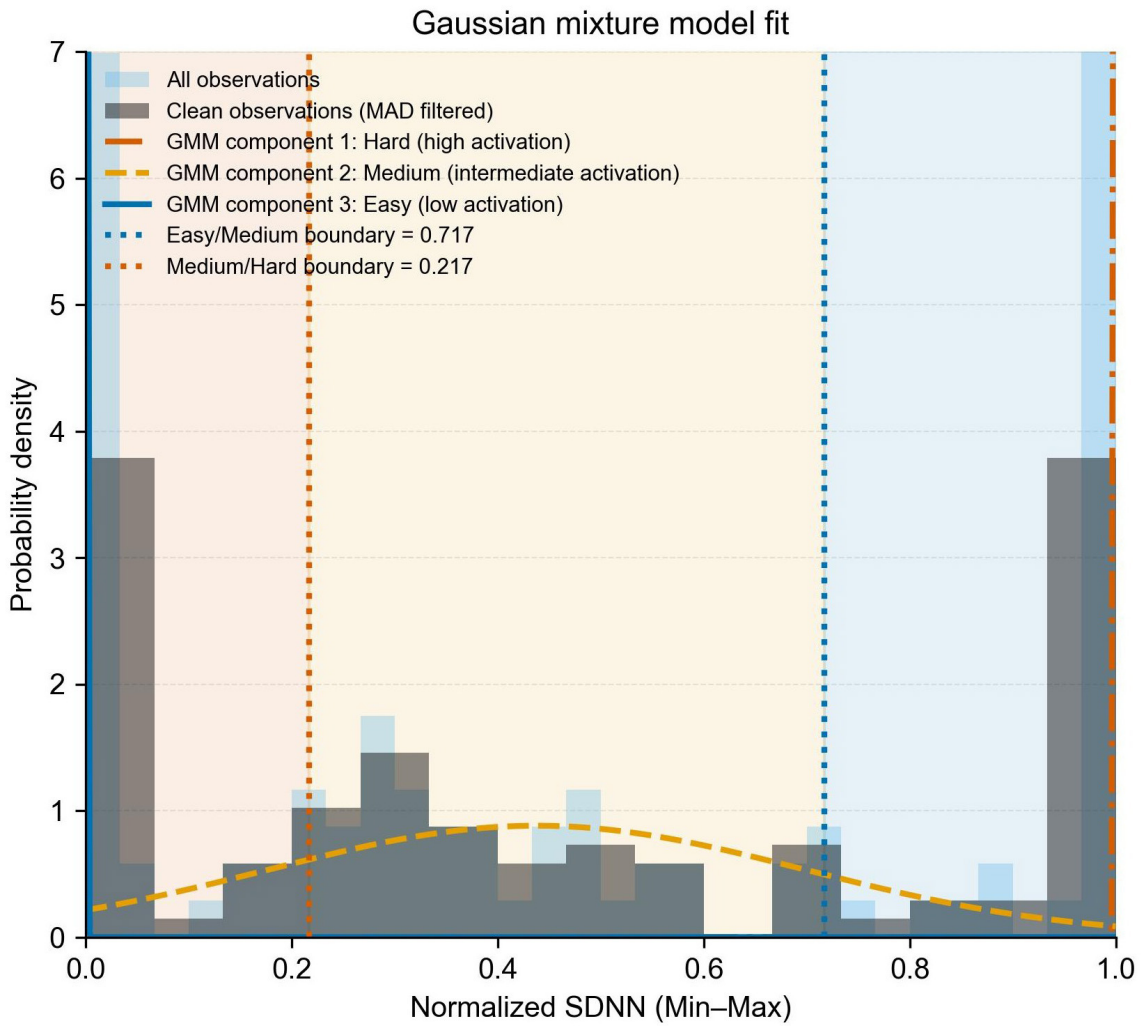
AeroStim system interface. Starting from the top left corner and proceeding clockwise, the interface hosts the following tasks: Aircraft Attitude Stroop Test, System Monitoring, Tracking, N-Back, Energy, Resource Management, Communication, Color Word Stroop Test.

Aerostim's interface is organized into several task modules designed to elicit cognitive processes. Two modules serve as primary tasks: the Tracking Task, which requires maintaining a dynamically moving crosshair within a target area using a joystick, and the System Monitoring Task, which requires continuous monitoring of visual cues and timely identification of anomalies. These tasks place sustained visuomotor and attentional demands. Both tasks mirror the corresponding subtasks of the MATB-II.

All remaining modules are classified as secondary tasks. These introduce increased simultaneous cognitive demands, divided attention, interference, and time pressure and have also been associated with stress-related responses in operational and laboratory settings (Staal, 2004). The N-back task targets working memory, requiring participants to determine whether the current digit corresponds to the one presented N steps earlier; although part of the AeroStim environment, it was not used in the present study. The Aircraft Attitude control task simulates aircraft instability and requires corrective actions in the presence of misleading signals. Exposure to disorientating stimuli in pilots has been associated with increased perceived stress and effort when disoriented by conflicting inputs (Bustamante-Sánchez and Clemente-Suárez, 2020). The Stroop test introduces interference by presenting incongruent combinations of colors and words; when administered in controlled conditions, it has been shown to increase perceived mental stress (Palanisamy et al., 2013). The communication task simulates radio exchanges in which participants respond only when their callsign (NASA504) is called; it requires selective auditory attention and response inhibition, particularly under multitasking conditions (Pontiggia et al., 2024). The resource management task requires adjusting the flow between interconnected tanks to maintain stable levels in the main tanks, accounting for capacity differences and flow constraints. Sustained cognitive demands under time pressure have been associated with increase perceived effort and autonomic activation (Delliaux et al., 2019).

An Energy bar, starting at its highest value, irreversibly decreases over time and task-related errors and omissions, providing a cumulative indicator of performance degradation during the session and a timing pressure load on the subject. In the present study, the depletion of the energy bar led to the early termination of the exercise.

### 2.3 Calibration of exercise difficulty and self-report

The Bedford Workload Scale (BWS) is a unidimensional subjective scale for assessing mental workload, developed at the Royal Aerospace Establishment in Bedford, UK, and formalized by Roscoe and Ellis (1990). The scale comprises 10 levels, ranging from negligible load (level 1) to mission abandonment (level 10), and is based on a hierarchical decision tree that guides the operator in estimating their residual capacity during task execution (Figure 2).

**Figure 2:**
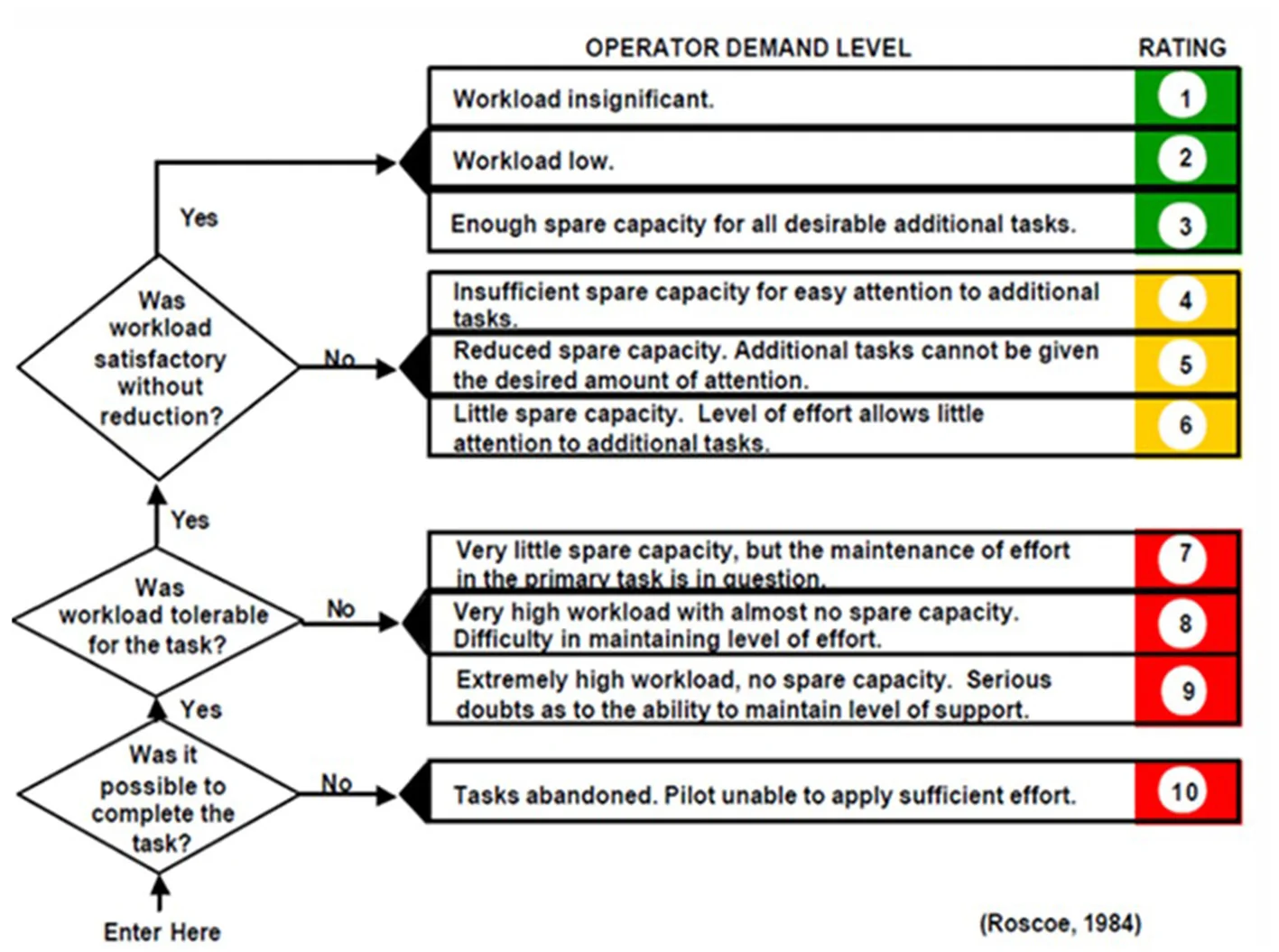
NASA-Bedford Workload Scale.

In this study, the BWS serves a dual role: (i) it was administered to participants as a self-assessment measure; (ii) it was used as a framework to define and standardize the difficulty levels of the experimental exercises.

A Visual Analog Scale (VAS) was used for assessed perceived stress. It is a 0–10 rating scale, where lower values indicate no stress and higher values indicate increased stress. VAS measures are widely used and validate for capturing subjective stress (Lesage and Berjot, 2011).

### 2.4 Experimental protocols and operational contexts

Two operational contexts were defined based on task structure and the presence of additional concurrent demands. In the Workload Only (WO) context, participants performed only the primary tasks, whereas in the Stress Oriented (SO) context, secondary tasks introducing divided attention, interference and time pressure, known to function as potential stressors (Staal, 2004). Participants were randomly assigned to WO (*n* = 20) or SO (*n* = 22) and completed a single four-exercise protocol with each exercise lasting up to 6 mins and terminate early in case of energy depletion (see Section 2.2).

The first three exercises represented three distinct difficulty ranges: low (1–3), medium (4–6), and high (8–9). The fourth exercise was set to the maximum difficulty level (10) and was designed to induce a performance breakdown. Each trial began with a 3-min resting period to establish baseline physiological parameters, followed by the exercises and, at the end of each exercise, the collection of self-reported measures. These three exercises were not separated by physiological rest periods to reproduce a situation of variable and continuous workload, as recommended in studies of autonomic dynamics under prolonged cognitive tasks (e.g., Fairclough and Houston, 2004). A second 3-min rest separated the third and fourth exercises: this was necessary to reset the physiological condition before assessing the response to the final exercise. Statistical comparisons were performed to ensure that the two rest periods provided comparable baseline physiological parameters. Because the same participants were assessed in both rest conditions, comparisons were performed using paired statistical tests. The normality of each HRV metric distribution was assessed separately for first and second baseline. Although normality was evaluated, none of the HRV metrics showed a normal distribution in both baseline conditions; therefore, all comparisons were performed using the Wilcoxon signed-rank test. To account for multiple comparisons between HRV metrics, *p*-values were corrected using the False Discovery Rate (FDR) Benjamini–Hochberg procedure. In addition to statistical significance, effect sizes were calculated using the rank-biserial correlation to quantify the magnitude and direction f baseline differences. Full statistical details are reported in the Supplementary Figure S1 and Supplementary Table S1. The entire session lasted approximately 30 mins. The order of the first three exercises was randomized to control for sequence effects, whereas the fourth task was always presented at the end to avoid carryover effects that could alter the physiological response in the first three exercises (McCarley, 2025) (Figure 3).

**Figure 3:**
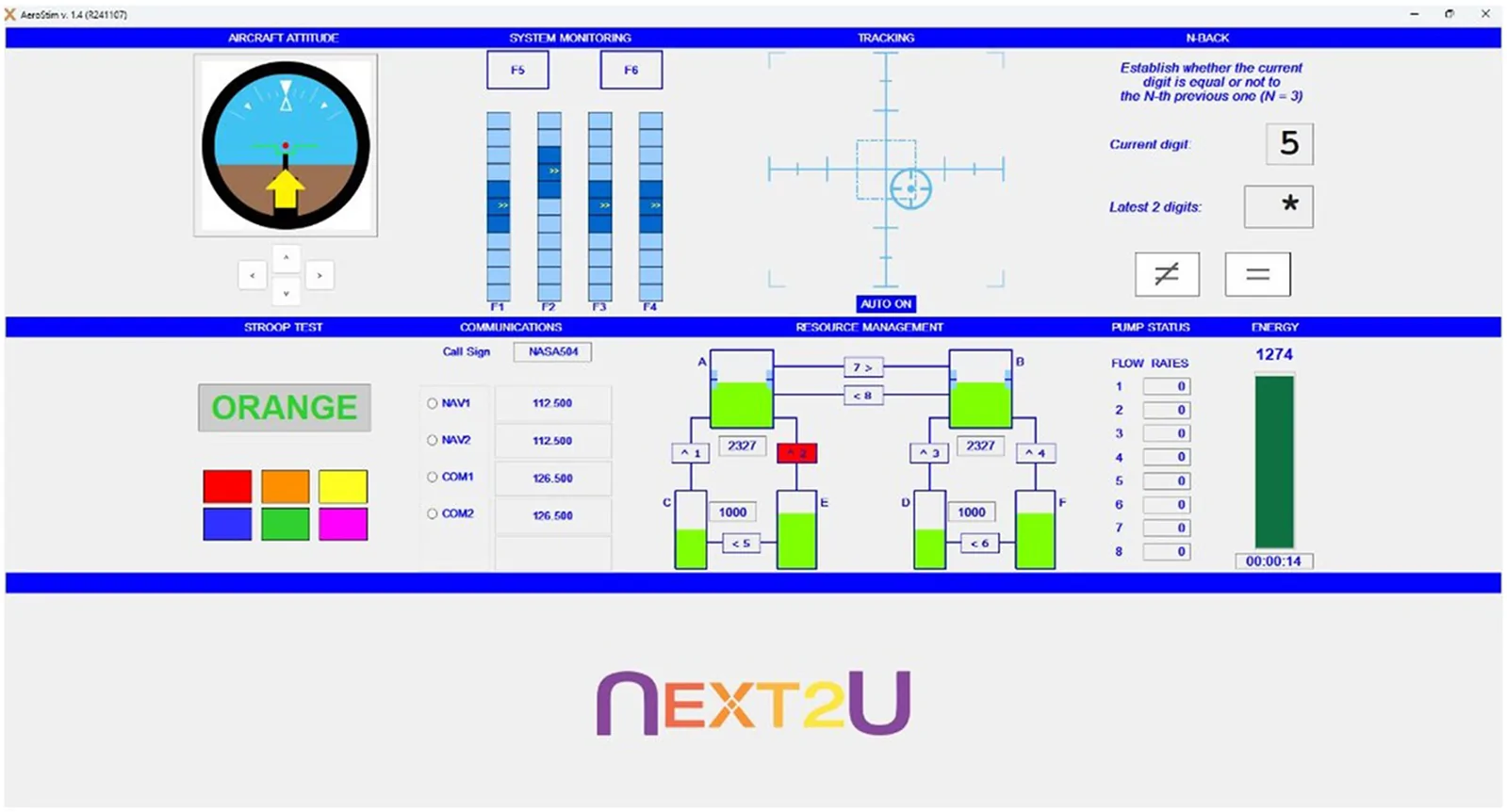
Trial exercises.

Because exercise termination depends on the energy depletion, its effect on exercise duration was characterized. The Supplementary Table S2 (WO) and Supplementary Table S3 (SO) report, for each participant and exercise, the difficulty level, duration, final energy value, and R-peak count. Each exercise was treated as an epoch, and shortened epochs were retained. Because epoch duration varied across exercises, no duration weighting was applied; therefore, potential effects of unequal epoch length could not be excluded. The corresponding distribution of epoch durations across exercises and subjects are shown in Supplementary Figure S2 (WO) and Supplementary Figure S3 (SO).

Exercises were constructed by combining, in a controlled manner, the tasks available in the AeroStim environment, selected according to the assigned operational context. Difficulty levels were defined a priori with reference to the BWS and reflected the presumed workload. Presumed workload modulation was achieved by adjusting task parameters—stimulus density and frequency, presentation speed, response time, and for tracking cursor reactivity and noise—whose increase, combined with a reduced response window, systematically heightened attentional demands and consequently cognitive workload and/or induced stress. Detailed parameter configurations for each level and context are provided in the Supplementary Figures S4, S5 and Supplementary Tables S4, S5.

### 2.5 Procedure and measurements

Prior to participation, all subjects provided written informed consent for data acquisition and processing in accordance with the General Data Protection Regulation (GDPR). All experiments were conducted in a quiet, temperature-controlled laboratory. Participants completed a familiarization session 1–2 days before the experiment to ensure full understanding of task requirements and interaction modalities. During the experimental session, physiological, behavioral, and subjective data were collected. Physiological signal was continuously recorded throughout the session, while performance metrics were extracted from AeroStim system logs. At the end of each exercise, subjective ratings were obtained using the NASA Bedford Workload Scale administered digitally.

The analyses reported in this study focus exclusively on HRV indices derived from electrocardiograpy and on performance metrics extracted from AeroStim logs.

#### 2.5.1 Recording and analysis of heart rate variability (HRV)

The Interbeat Intervals (IBI) were recorded and extracted using an EqVital sensorized digital T-shirt (Equivital Ltd, n.d.), a wearable system for real-time multiparameter physiological monitoring. The garment integrates textile electrodes in a two-lead ECG configuration and a Sensor Electronics Module (SEM) that wirelessly acquires the signal at 256 Hz and automatically derives RR intervals. Although the proprietary algorithm used for IBI detection is not publicly disclosed, the Equivital EQ02 has been validate against reference Holter ECG, showing high accuracy in R-peak detection and HRV estimation (Akintola et al., 2016).

Signal acquisition was managed via HIRA Next2U's software (https://www.next2u-solutions.com), which synchronizes multimodal data streams using a unified timestamp, providing a common temporal reference for the alignment of physiological and behavioral data. The synchronization architecture was designed to minimize temporal latency between streams; given the temporal scale of the present analyses, any residual synchronization delay was considered negligible. The resulting IBI series served as the basis for computing the HRV indices.

##### 2.5.1.1 IBI signal preprocessing

Signal preprocessing was conducted on the entire time series IBI signal for each participant. It consisted of four sequential steps: (i) removal of physiological outliers using exclusion thresholds in the range 300–2,000 ms (Sammito et al., 2024), replacing excluded values with missing values (NaNs); (ii) linear interpolation of NaNs; (iii) detection and removal of ectopic beats using the Malik criterion (*RR*_i_−*RR*_i − 1_|/*RR*_i − 1_ > 0.20) (Kamath and Fallen, 1995), replacing identified beats with NaNs; and (iv) a second linear interpolation to preserve signal continuity (Choi and Shin, 2018).

Quality-control metrics were computed for each participant, including the number of detected outliers, ectopic beats, and corrected intervals, together with the percentage of corrected beats relative to the total number of IBI. Across participants, the proportion of corrected intervals remained low in both experimental contexts (WO: 0.79% ± 1.02%; SO: 1.11% ± 1.68%), indicating overall a good signal quality. Detailed quality-control statistics for individual participants are reported in Supplementary Table S6.

##### 2.5.1.2 HRV indices

HRV was analyzed using time- and frequency-domain indices derived from the IBI series. Before index extraction, the IBI signal was segmented into epochs corresponding to the entire durations of the active phase of exercises, excluding rest periods and questionnaire completion intervals. HRV metrics were extracted from each phase in accordance with recommendations of the Task Force for the Analysis of Metrics in the Frequency Domain which require windows of at least 2 mins duration (Task Force of the European Society of Cardiology the North American Society of Pacing Electrophysiology, 1996).

Time-domain indices quantify statistical proprieties of the NN interval sequence ${\left\{NN_{i}\right\}}_{i=1}^{N}$ where {*NN*_*i*_} denotes the *i*-th successive interbeat interval in milliseconds. The standard deviation of NN intervals (SDNN) was computed as:


$$SDNN=\;\surd \frac{1}{N-1}\sum_{i=1}^{N}{\left(NN_{i}-\bar{NN}\right)}^{2}$$

where $\bar{NN}$is the mean of all *NN* intervals in the epoch. SDNN reflects the total variability of the IBI series attributable to all cyclical components of autonomic activity, and is therefore sensitive to both sympathetic and parasympathetic contributions across a range of timescales (Kleiger et al., 2005; Shaffer et al., 2014).

The root mean square of successive differences (RMSSD) was computed as:


$$RMSSD=\;\surd \frac{1}{N-1}\sum_{i=1}^{N-1}{\left(NN_{i+1}-{\bar{NN}}_{i}\right)}^{2}$$

RMSSD quantified beat-to-beat variability driven by rapid vagal modulation of cardiac rhythm, and is considered the most robust time-domain index of parasympathetic activity due to its independence from respiratory confounds affecting longer-term variance estimates (Thayer and Lane, 2000; Shaffer et al., 2014).

The proportion of successive NN interval differences exceeding 50 ms (pNN50) was computed as:


$$\begin{array}{l} pNN50=\;\frac{\left|\left\{i\;:|NN_{i+1}-NN_{i}|>50ms\right\}\right|}{N-1}\;x\;100 \end{array}$$

pNN50 provides a complementary index of high-frequency parasympathetic modulation and has been shown to correlate strongly with RMSSD while offering greater sensitivity to vagal tone at rest (Mietus et al., 2002; Shaffer and Ginsberg, 2017).

SD1 and SD2 were derived from the Poincaré plot of the NN series, defined as:


$$SD1=\frac{SDSD}{\surd^{2}};SD2=\;\sqrt{2SDNN^{2}-SD1^{2}}$$

Where SDSD denotes the standard deviation of successive NN differences. SD1 quantifies short-term, beat-to-beat variability and is mathematically equivalent to RMSSD/$\surd^{2}$, reflecting rapid parasympathetic modulation. SD2 captures longer-term variability encompassing both sympathetic and parasympathetic contributions to autonomic regulation (Brennan et al., 2001; Shaffer and Ginsberg, 2017).

Frequency-domain indices were estimated by computing the power spectral density (PSD) of the IBI series using the “*get_frequency_domain_features*” function from the Python library *hrv-analysis*. It applies Welch's method with cubic interpolation of RR intervals (resampled at 4 Hz), mean detrending and PSD estimation using a 256-sample Hamming window with 50% overlap. Spectral power in each band was obtained by integrating the PSD over the corresponding frequency range:


$$\begin{array}{l} P_{band}=\int_{f_{low}}^{f_{high}}S\left(f\right)df \end{array}$$

where *S*(*f*)is the estimated power spectral density in ms^2^/Hz. The high-frequency band (HF: 0.15–0.40 Hz) was used as a marker of cardiac vagal tone, as oscillations in this range are predominantly mediated by the respiratory sinus arrhythmia reflex and reflect direct parasympathetic modulation of the sinoatrial node (Malik et al., 1996). Consequently, reductions in HF power under cognitive load or stress are interpreted as withdrawal of parasympathetic tone, consistent with the neurovisceral integration model (Thayer et al., 2012). The low-frequency band (LF: 0.04–0.15 Hz) was also considered. LF power reflects a mixture of sympathetic and parasympathetic activity, baroreflex gain modulation, and slow respiratory influences, and should therefore not be interpreted as a selective marker of sympathetic outflow (Billman, 2011; Reyes del Paso et al., 2013).

The LF/HF ratio:


$$\begin{array}{l} \frac{LF}{HF}=\;\frac{P_{LF}}{P_{HF}} \end{array}$$

was included as a descriptive index of the relative distribution of autonomic spectral power across frequency bands. In line with current recommendations (Billman, 2011), the LF/HF ratio was not interpreted as a direct index of sympathetic–parasympathetic balance—given that LF power reflects a mixture of both autonomic branches and is strongly influenced by baroreflex gain and respiratory patterns—but was considered in conjunction with the remaining HRV indices to provide a more comprehensive characterization of autonomic dynamics across different operational contexts.

Non-linear indices were computed to capture the complexity and irregularity of the NN series that are not accessible through linear analyses, and that have been shown to be differentially sensitive to autonomic dysregulation and cognitive stress (Melillo et al., 2011; Laborde et al., 2017).

Sample Entropy (SampEn) was computed following Richman and Moorman (2000):


$$\begin{array}{l} SampEn\left(m,\;r,\;N\right)=\;-ln\frac{A}{B} \end{array}$$

Where *B* denotes the number of template pairs of length *m* whose Chebyshev distance is less than or equal to the tolerance *r*, and *A* denotes the corresponding count for templates of length *m*+*1*. Parameters were set to *m* = 2 and *r* = 0.25 × SD(NN), in accordance with established conventions (Richman and Moorman, 2000; Lake et al., 2002). SampEn quantifies the conditional probability that two sequences similar for *m* points remain similar for one additional point; higher values indicate grater signal irregularity and complexity. Decreases in SampEn have been associated with increased sympathetic dominance, mental stress, and reduced autonomic adaptability (Melillo et al., 2011; Byun et al., 2019).

Fuzzy Entropy (FuzzyEn) was computed following Chen et al. (2009):


$$\begin{array}{l} FuzzyEn\left(m,\;r,\;N\right)=\;-ln\frac{\Phi^{m+1}}{\Phi^{m}} \end{array}$$

Where Φ^*m*^ represents the average fuzzy membership across all template pairs of length *m*, computed using the exponential fuzzy function $\mu \left(d\right)=\exp\left[-{\left(\frac{d}{r}\right)}^{n}\right],$ with *d* the Chebyshev distance between baseline-removed template vectors. Parameters were set to *m* = 2, *r* = 0.20 × SD(NN), and *n* = 2. Compared to SampEn, FuzzyEn replaces the hard binary boundary with a continuous similarity function, yielding more stable estimates with reduced dependence on data length and parameter selection (Chen et al., 2009).

Permutation Entropy (PermEn) was computed following Bandt and Pompe (2002):


$$\begin{array}{l} PermEn\left(m,\tau \;\right)=\;-\underset{\pi }{\sum }p\left(\pi \right)\ln\text{ p}\left(\pi \right) \end{array}$$

Where the sum is taken over all *m!* possible ordinal patterns π of embedding dimension *m, p(*π*)* denotes the relative frequency of each pattern in the time series, and τ is the time delay. The normalized from PermEn/ln(m!) ε [0, 1] was adopted to allow comparison across embedding dimensions. Parameters were set to *m* = 3, and τ = 1. PermEn captures the temporal ordering complexity of the NN series based on the rank structure of local amplitude patterns rather than their absolute values, rendering it robust to non-stationary fluctuations and amplitude artifacts (Bandt and Pompe, 2002; Bian et al., 2012). Higher PermEn values indicate greater irregularity in the sequential beat-to-beat pattern.

The correlation dimension D2 was estimated using Grassberger-Procaccia algorithm as implemented in the *nolds* Python library (Grassberger and Procaccia, 1983), with an embedding dimension of 10. D2 quantifies the fractal dimensionality of the reconstructed attractor in phase space: lower values reflect a more deterministic and low-dimensional dynamic structure, whereas higher values indicate greater stochastic complexity. Reduction in D2 during acute stress have been interpreted as a loss of non-linear complexity in autonomic cardiac control, consistent with the complexity loss hypothesis of physiological aging and disease (Lipsitz and Goldberger, 1992).

#### 2.5.2 Performance metrics

Evaluation of the behavioral performance was conducted using only metrics extracted from the primary tasks, as they were present in both experimental contexts (Workload-Only, Stress-Oriented), thus allowing direct and consistent comparisons between the two conditions. Secondary tasks were excluded from performance analyses because they were specifically introduced to induce stress, rather than providing a reliable measure of performance.

For the Tracking task, the mean distance of the cursor from the target area (*adt_mean*) was used as an indicator of the precision of continuous motor control. This output parameter has been repeatedly shown to increase with increasing cognitive demand and to reflect the degradation of sensorimotor resources under mental load (Pontiggia et al., 2024). For the System Monitoring task, four complementary behavioral indicators were considered: percentage of missed responses (*m_SyS*), percentage of incorrect responses (*w_SyS*), percentage of correct responses (*c_SyS*), and mean response time (*mRT_SyS*). This multi-metric approach follows the recommendation to assess sustained attention through both accuracy and response latency, since these dimensions can vary independently under different workload and stress conditions (Warm et al., 2008). Missed and incorrect responses are standard vigilance indices sensitive to cognitive overload and fatigue, while correct rate and mean reaction time describe the operator's speed-accuracy profile (See et al., 1995). The combined use of accuracy and response time is also supported by the speed-accuracy tradeoff framework, according to which reaction time and error rate represent complementary dimensions of cognitive performance and may be differently affected by workload, stress, task demands, and response strategies (Heitz, 2014). Performance metrics were calculated over the entire duration of each exercise.

#### 2.5.3 Intra-subject normalization

In order to remove individual differences in the absolute values of HRV metrics and to make physiological profiles comparable between participants, each metric was normalized with the intra-subject Min-Max method, using as a reference the minimum and maximum values of the individual participant's distribution across all experimental phases, global Min-Max:


$$\begin{array}{l} x_{norm}=\;\frac{x-min}{\max-\;min} \end{array}$$

This approach produces values in the interval [0, 1] and allows each observation to be expressed in relative terms with respect to the individual physiological range, without assuming normality in the distribution of residuals.

#### 2.5.4 Definition of physiological activation ranges

Physiological activation levels was defined a priori into three discrete ranges—*Easy, Medium, Hard*—consistent with inverted-U models of arousal (Hebb, 1955). The number of regimes derives from a theoretical hypothesis and was not estimated from the data; the GMM was used exclusively to identify the boundaries between the three levels based on normalized within-subject SDNN values. The SDNN was chosen as a global index of HRV, sensitive to tonic modulations of autonomic activity associated with sustained changes in arousal and cognitive load (Damoun et al., 2024).

To test the plausibility of the three-regime structure, GMMs with 1–6 components were compared using AIC and BIC. The three-component solution was retained for theoretical reasons, and the BIC provided further support for the parsimonious choice (Supplementary Table S7). Boundary stability was assessed through a sensitivity analysis with different normalizations (Min–Max, *Z*-score, robust *Z*-score, and percentile). Classifications showed 98.4%−100% agreement, indicating that the tripartite classification is robust to normalization. Because the Min–Max normalization uses the entire session, the boundaries reflect the distribution of SDNN values from the individual recording and not generalizable physiological thresholds. A real-time application would therefore require a brief initial calibration to estimate subject parameters, followed by prospective classification based on the pre-trained GMM.

Within this framework, the GMM identified three ranges (0–0.217; 0.217–0.717; 0.717–1.00) representing relative differences in HRV. Labels were assigned a posteriori based on SDNN mean values: the highest range (0.717–1.00), indicative of greater autonomic flexibility and parasympathetic predominance, was classified as Easy; the intermediate range (0.217–0.717) as Medium; and the lowest range (0–0.217), associated with reduced HRV and greater sympathetic activation, as Difficult (Arakaki et al., 2023; Damoun et al., 2024).

The GMM was fitted to the aggregated data from the two task contexts (WO and SO), with three Gaussian components (full covariance) and 20 random initializations. Outliers were removed using a modified *Z*-score (threshold > 3.5; Leys et al., 2013). Regime boundaries were defined as intersection points between Gaussian densities, and their stability was assessed through bootstrapping (200 resamples), yielding standard deviations of ±0.079 (Easy/Medium) and ±0.013 (Medium/Difficult).

The distribution of observations across the three regimes was similar in the two contexts: WO (Easy 25.7%, Medium 43.2%, and Difficult 31.1%) and SO (Easy 23.5%, Medium 42.0%, and Difficult 34.6%). Figure 4 shows the distribution of normalized SDNN values, the Gaussian components, and the estimated boundaries.

**Figure 4:**
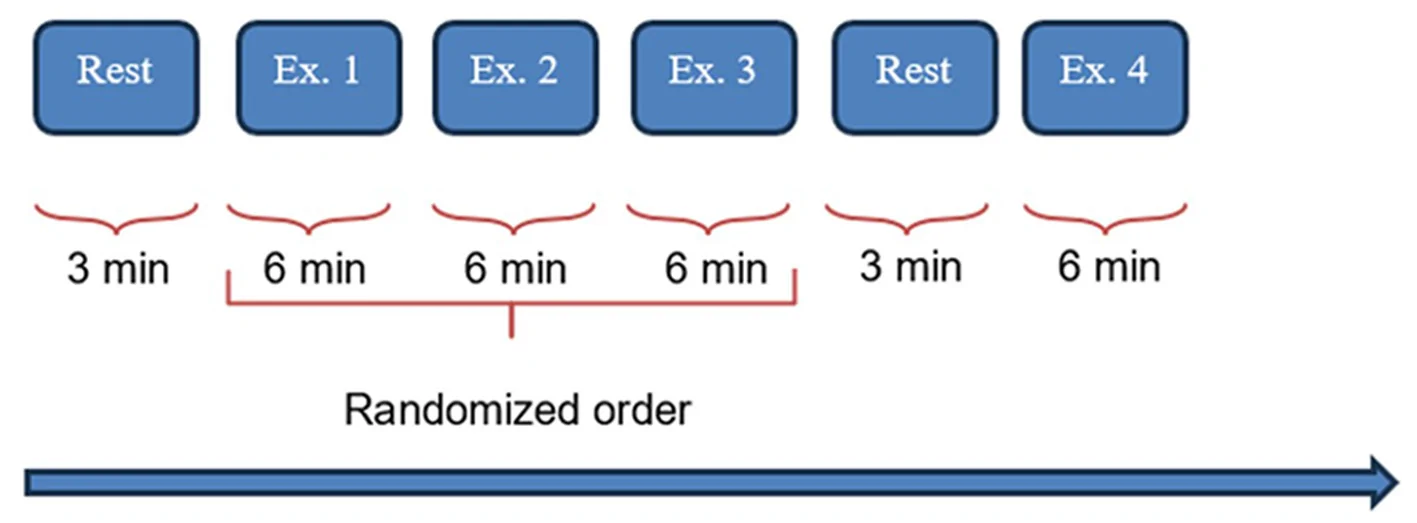
Distribution of the normalized SDNN values. The colored curves indicate the Gaussian components estimated by the model. The intersections between adjacent components, represented by dashed vertical lines, defined the thresholds used to classify autonomic activation levels.

#### 2.5.5 Statistical analysis

Statistical analyses were organized into three sequential levels. At Level 1, difficulty-performance and HRV-performance associations were assessed using Spearman's rank correlation coefficient (ρ), with confidence intervals, permutation-based inference, and by Benjamini–Hochberg correction. At Level 2, performance differences across activation ranges within each operational context were examined using the Kruskal–Wallis test with Bonferroni *post-hoc* comparisons when appropriate. At Level 3, performance was compared between operational contexts (*WO*; *SO*) within each activation range using the Mann–Whitney *U-*test, with Benjamini–Hochberg correction. Two exploratory analyses were performed. First, the effects of task-difficulty ranges (*Easy, Medium*, and *High*) on perceived workload and stress were examined separately for each operational context. Second, the impact of the Energy Bar on epoch duration was characterized, including the proportion of epochs meeting the minimum recording-duration requirements reported in the HRV literature for each metric. Two sensitivity analyses were additionally conducted. First, to assess the robustness of HRV estimates to variable epoch duration, all HRV metrics were recalculated using fixed 180 s window and compared with estimates from full-length epochs. The 180 s duration was selected for commonly recommended minimum durations for time- and frequency-domain indices (Task Force of the European Society of Cardiology the North American Society of Pacing Electrophysiology, 1996; Shaffer and Ginsberg, 2017). Second, to determinate whether SDNN-performance associations depended on the SDNN-based GMM stratification, correlations were recomputed using task-difficulty ranges as an alternative stratification criterion, applying the same inference and correction procedures. All analyses were conducted in Python (Python Software Foundation, Wilmington, DE, United States).

##### 2.5.5.1 Window-length sensitivity analysis

To assess the sensitivity of HRV estimates to window duration, HRV metrics were recalculated using the entire phase and a shorter fixed window of 180 s (or shorter windows were assumed to be the entire window). For each metric, estimates obtained from the shorter windows were compared to the estimate for entire phase.

##### 2.5.5.2 Correlation analyses

Correlations between task-difficulty and performance, and between HRV indices and performance, were evaluated using Spearman's rank correlations coefficient (ρ), a non-parametric approach selected because several physiological and behavioral variables deviated from normality. The difficulty-performance analysis was conducted as a preliminary analysis to assess whether the predefined difficulty manipulation was reflected in behavioral performance. HRV-performance correlations were subsequently evaluated. Analysis were conducted separately within each activation range (*Easy, Medium*, and *Hard*) and operational context (*WO*; *SO*).

To reduce within-range dependence arising from repeated observations from the same participant, repeated observations from the same participant, repeated observations within each range were aggregated using the median prior to analysis. This approach was preferred over linear mixed-effects modeling because only a limited proportion of participants contributed more than one observation per range, resulting in an insufficient repeated-measures structure to reliably estimate subject-specific random effects (Bland and Altman, 1996). The distribution of observations (Obs) across activation ranges before and after aggregation is reported in Table 1.

**Table 1:** Distribution of observations across activation ranges in the WO and SO contexts.

Activation range	Condition	Original obs	Obs per participant (median/max)	Obs from same subject	Obs after aggregation
Easy	WO	19	1.0/2	5 (35.7%)	14
	SO	19	1.0/2	3 (18.8%)	16
Medium	WO	28	2.0/3	10 (55.6%)	18
	SO	29	2.0/3	11 (61.1%)	18
Hard	WO	23	1.0/2	6 (35.3%)	17
	SO	27	1.0/3	8 (42.1%)	19

For each HRV-performance pair, statistical significance was assessed using the asymptotic Spearman *p*-value and a permutation-based test (*n* = 5,000 permutations), in which participant labels were randomly shuffled to generate an empirical null distribution of correlation coefficients. To quantify the uncertainty of the correlation estimates, 95% confidence intervals were estimated via participant-level bootstrap resampling (5,000 resamples, percentile method) applied after within-subject median aggregation. Given the small number of participants contributing to individual activation-range cells, correlation coefficients and their confidence intervals were interpreted with caution. Given the large number of tests performed, all *p*-values were adjusted for multiple comparisons using the Benjamini–Hochberg false discovery rate correction (FDR, α = 0.05; Benjamini and Hochberg, 1995). For each correlation, ρ, its 95% confidence interval, raw *p*-value, permutation *p*-value, and corresponding FDR-adjusted *p*-values are reported (Tables 2–5).

**Table 2:** Spearman correlations between task difficulty and performance metrics across physiological activation ranges in the Workload-Only condition.

Activation Range	Metric	ρ	CI (95%)	p_FDR	ρ perm	*p* val perm	p_FDR-perm
Easy	c_SyS	−0.70	(−0.92, −0.23)	0.21	−0.70	<0.01	0.21
	m_SyS	0.67	(0.17, 0.91)	0.21	0.67	<0.01	0.21
	adt_mean	0.69	(0.24, 0.94)	0.21	0.69	<0.01	0.21
Medium	m_SyS	0.51	(0.03, 0.78)	0.47	0.67	<0.01	0.40
	adt_mean	0.64	(0.21, 0.90)	0.21	0.64	<0.01	0.21
Hard	adt_mean	0.71	(0.20, 0.96)	0.21	0.71	<0.01	0.21

For each significant association (p_raw <0.05), Spearman's ρ, its 95% confidence interval (CI95%) obtained via participant-level bootstrap resampling, permutation ρ (ρ perm) and p-value (p val perm), and FDR-adjusted p-values (p_FDR, p_FDR-perm) are reported. mRT_SyS, mean reaction time; c_SyS, percentage of correct responses; w_SyS, percentage of incorrect responses; m_SyS, omission rate (System Monitoring task); adt_mean, mean cursor distance from target (Tracking task).

**Table 3:** Spearman correlations between task difficulty and performance metrics across physiological activation ranges in the Stress-Oriented condition.

Activation range	Metric	ρ	CI (95%)	p_FDR	ρ perm	*p* val perm	p_FDR-perm
Easy	c_SyS	−0.53	(−0.85, 0.01)	0.30	−0.53	0.03	0.33
	w_SyS	0.65	(0.23, 0.88)	0.11	0.65	0.01	0.21
	m_SyS	0.55	(0.02, 0.87)	0.30	0.55	0.02	0.26
	adt_mean	0.78	(0.35, 0.94)	0.11	0.78	<0.01	<0.01
Medium	c_SyS	−0.76	(−0.92, −0.43)	0.01	−0.76	<0.01	<0.01
	w_SyS	0.49	(0.01, 0.85)	0.30	0.49	0.03	0.30
	m_SyS	0.73	(0.38, 0.90)	0.02	0.73	<0.01	<0.01
	adt_mean	0.95	(0.80, 0.98)	<0.01	0.95	<0.01	<0.01
Hard	c_SyS	−0.46	(−0.76, 0.01)	0.30	−0.46	0.048	0.33
	adt_mean	0.83	(0.53, 0.95)	<0.01	0.83	<0.01	<0.01

For each significant association (p_raw <0.05), Spearman's ρ, its 95% confidence interval (CI95%) obtained via participant-level bootstrap resampling, permutation ρ (ρ perm) and p-value (p val perm), and FDR-adjusted p-values (p_FDR, p_FDR-perm) are reported. mRT_SyS, mean reaction time; c_SyS, percentage of correct responses; w_SyS, percentage of incorrect responses; m_SyS, omission rate (System Monitoring task); adt_mean, mean cursor distance from target (Tracking task).

**Table 4:** Spearman correlations between HRV index and performance metrics across physiological activation ranges in the Workload-Only condition.

Activation range	HRV index	Performance	ρ	CI 95%	p_FDR	ρ perm	*p* val perm	p_FDR-perm
Easy	LF	adt_mean	0.53	(0.03, 0.81)	0.69	0.53	0.05	0.67
		c_SyS	−0.55	(−0.83, −0.08)	0.69	−0.55	0.05	0.67
		m_SyS	0.56	(0.08, 0.83)	0.69	0.56	0.04	0.67
Medium	SDNN	adt_mean	0.60	(0.16, 0.87)	0.47	0.60	0.05	0.42
		m_SyS	0.47	(−0.03, 0.79)	0.69	0.47	0.04	0.67
	LF	m_SyS	−0.46	(−0.74, −0.02)	0.69	−0.46	0.05	0.67
	SD2	adt_mean	0.55	(0.14, 0.82)	0.59	0.55	0.01	0.54
Hard	SDNN	w_SyS	0.65	(0.27, 0.92)	0.47	0.65	0.04	0.42
	LF/HF	mRT_SyS	−0.53	(−0.85, −0.01)	0.69	−0.53	0.03	0.67
	SampEn	c_SyS	0.58	(0.15, 0.84)	0.59	0.58	0.01	0.54

The table includes only HRV indices that showed at least one significant association with performance metrics (p_raw <0.05). For each significant correlation, Spearman's ρ, its 95% confidence interval (CI95%) obtained via participant-level bootstrap resampling, permutation-based ρ (ρ perm) and p-value (p val perm), and FDR-adjusted p-values (p_FDR, p_FDR-perm) are reported. Performance metrics: mRT_SyS, mean reaction time; c_SyS, percentage of correct responses; w_SyS, percentage of incorrect responses; m_SyS, omission rate (System Monitoring task); adt_mean, mean cursor distance from target (Tracking task).

**Table 5:** Spearman correlations between HRV index and performance metrics across physiological activation ranges in the Stress-Oriented condition.

Activation range	HRV index	Performance	ρ	CI (95%)	p_FDR	ρ perm	*p* val perm	p_FDR-perm
Easy	LF	mRT_SyS	0.65	(0.23, 0.87)	0.24	0.64	0.013	0.39
		c_SyS	−0.51	(−0.85, −0.08)	0.40	−0.51	0.06	0.44
		m_SyS	0.51	(0.08, 0.86)	0.40	0.50	0.59	0.44
Medium	RMSSD	mRT_SyS	−0.69	(−0.89, −0.32)	0.12	−0.69	0.03	0.27
		m_SyS	−0.52	(−0.86, 0.11)	0.40	−0.52	0.02	0.39
	LF	mRT_SyS	−0.47	(−0.82, 0.06)	0.40	−0.47	0.06	0.45
	pNN50	mRT_SyS	−0.62	(−0.83, −0.24)	0.24	−0.62	<0.01	0.39
	SD1	mRT_SyS	−0.69	(−0.89, −0.31)	0.12	−0.69	0.03	0.27
		m_SyS	−0.52	(−0.87, 0.02)	0.40	−0.52	0.02	0.39
	SD2	c_SyS	−0.51	(−0.74, −0.11)	0.40	−0.51	0.036	0.44
		m_SyS	0.47	(0.03, 0.72)	0.40	0.47	0.049	0.44
	FuzzyEn	adt_mean	−0.49	(−0.83, 0.01)	0.40	−0.49	0.04	0.44
		mRT_SyS	−0.56	(−0.83, −0.10)	0.40	−0.56	0.02	0.39
		c_SyS	0.49	(−0.04, 0.80)	0.40	0.49	0.05	0.44
		m_SyS	−0.63	(−0.86, −0.18)	0.24	−0.63	0.01	0.39
	PermEn	adt_mean	−0.57	(−0.86, −0.09)	0.39	−0.57	0.01	0.39
Hard	RMSSD	w_SyS	−0.46	(−0.79, 0.02)	0.40	−0.46	0.04	0.44
	HF	w_SyS	−0.47	(−0.80, −0.01)	0.40	−0.47	0.03	0.44
	LF	w_SyS	−0.52	(−0.87, −0.01)	0.40	−0.52	0.02	0.39
	SD1	w_SyS	−0.46	(−0.79, 0.02)	0.40	−0.46	0.04	0.44

The table includes only HRV indices that showed at least one significant association with performance metrics (p_raw <0.05). For each significant correlation, Spearman's ρ, its 95% confidence interval (CI95%) obtained via participant-level bootstrap resampling, permutation**-**based ρ (ρ perm) and p-value (p val perm), and FDR**-**adjusted p**-**values (p_FDR, p_FDR-perm) are reported. Performance metrics: mRT_SyS, mean reaction time; c_SyS, percentage of correct responses; w_SyS, percentage of incorrect responses; m_SyS, omission rate (System Monitoring task); adt_mean, mean cursor distance from target (Tracking task).

##### 2.5.5.3 Sensitivity analysis of SDNN-based stratification on SDNN correlations

To assess whether the observed associations between SDNN and performance depended on the SDNN-based GM stratification, the correlation analysis was repeated using task difficulty as an independent stratification criterion. Task difficulty levels were grouped according to the definition used by BWS into three ranges (*Easy*: 1–3; *Medium*: 4–6; *High*: 7–10). For each operational context within each range, Spearman correlations between SDNN and performance metrics were recalculated using the same permutation-based inference and Benjamini–Hochberg FDR correction described previously. The resulting correlation and significance coefficients were compared with those obtained using the physiological arousal ranges to assess the robustness of the results.

##### 2.5.5.4 Comparisons across activation ranges

Performance differences across activation ranges were examined separately for the *WO* and *SO* using the Kruskal–Wallis test (H), employed as an omnibus screening step to assess whether *post-hoc* comparisons were justified. Effect sizes were quantified by eta-squared (η^2^) and epsilon-squared (ε^2^). When a significant omnibus effect was detected (*p* < 0.05), pairwise *post-hoc* comparisons were conducted using Mann–Whitney *U*-tests with Bonferroni correction. Cliff's delta (δ) was reported as a non-parametric effect size measure and interpreted according to the following thresholds: negligible (|δ| <0.147), small (0.147 ≤ |δ| <0.330), medium (0.330 ≤ |δ| <0.474), and large (|δ| ≥ 0.474; Romano and Kromrey, 2006) (see Tables 6–8).

**Table 6:** Kruskal–Wallis test results for performance differences across physiological activation ranges in the Workload-Only condition.

Performance metric	*H*(2)	ρ	η^2^	ε^2^
mRT_SyS	4.780	0.091	0.039	0.037
c_SyS	5.920	0.051	0.055	0.052
w_SyS	3.235	0.198	0.017	0.016
m_SyS	6.403	0.040	0.062	0.058
adt_mean	5.047	0.080	0.042	0.040

H, Kruskal–Wallis statistic; df, 2 for all tests; η^2^, eta-squared effect size; ε^2^, epsilon-squared effect size. Statistically significant results are characterized by p <0.05. mRT_SyS, mean reaction time; c_SyS, percentage of correct responses; w_SyS, percentage of incorrect responses; m_SyS, omission rate (System Monitoring); adt_mean, mean cursor distance from target (Tracking task).

**Table 7:** *Post-hoc* pairwise comparisons (Dunn's test with Bonferroni correction) for performance across activation ranges in the Workload-Only condition.

Metric	Contrast	*p*	δ	Interpretation
m_SyS	Low vs. intermediate	0.039	0.36	Significantly lower m_SyS at intermediate
	Low vs. hard	n.s	–	–
	Intermediate vs. hard	n.s.	–	–

Statistically significant results are characterized by p <0.05, n.s., not significant; otherwise. p = Bonferroni corrected p-value. δ, Cliff's delta effect size. m_SyS, omission rate (System Monitoring).

**Table 8:** Kruskal–Wallis test results for performance differences across physiological activation ranges in the Stress-Oriented condition.

Performance metric	*H*(2)	ρ	η^2^	ε^2^
mRT_SyS	1.671	0.433	−0.004	−0.004
c_SyS	1.164	0.558	−0.010	−0.010
w_SyS	0.293	0.863	−0.021	−0.020
m_SyS	1.085	0.581	−0.011	−0.011
adt_mean	1.933	0.380	−0.0009	−0.0008

H, Kruskal–Wallis statistic; df, 2 for all tests; η^2^, eta-squared effect size; ε^2^, epsilon-squared effect size. No comparisons reached statistical significance (all p > 0.05).

##### 2.5.5.5 Between-condition comparisons

As an exploratory analysis, behavioral performance was compared between *WO* and *SO* conditions using Mann–Whitney *U*-tests with FDR. To avoid within-range dependence, repeated observations were aggregated at the participant level (subject median per range). If the previous analysis identified a specific activation range associated with the most favorable performance profile, comparisons between the *WO* and *SO* conditions were limited to that range. Conversely, if no optimal range emerged, the analysis proceeded by comparing all activation ranges. This approach allowed us to assess whether comparable levels of autonomic activation were associated with different behavioral outcomes across different workload and stress conditions. Cliff's delta (δ) was reported as the effect size, following the interpretation criteria described in Section 2.5.5.2 (see Table 9).

**Table 9:** Between-condition comparison of performance metrics at the physiological activation ranges.

Range	Metric	Workload-only Mean ±SD	Stress-oriented Mean ±SD	*U*	*p*	p_FDR	δ
Easy	mRT_SyS	2.055 ± 0.454	2.8971 ± 0.790	39.00	0.002	0.019	−0.65
	c_SyS	0.654 ± 0.186	0.737 ± 0.213	82.00	0.219	0.412	−0.26
	w_SyS	0.047 ± 0.022	0.018 ± 0.023	181.00	0.003	0.019	0.61
	m_SyS	0.299 ± 0.177	0.245 ± 0.205	130.50	0.453	0.567	0.16
	adt_mean	19.704 ± 19.0	10.997 ± 14.1	158.00	0.058	0.124	0.41
Medium	mRT_SyS	2.046 ± 0.60	2.753 ± 0.76	80.00	0.009	0.029	−0.50
	c_SyS	0.798 ± 0.20	0.771 ± 0.15	179.00	0.601	0.667	0.10
	w_SyS	0.043 ± 0.05	0.018 ± 0.03	245.00	0.008	0.029	0.51
	m_SyS	0.159 ± 0.17	0.208 ± 0.14	134.00	0.383	0.523	−0.17
	adt_mean	5.548 ± 5.75	6.769 ± 11.4	173.00	0.739	0.739	0.07
Hard	mRT_SyS	2.314 ± 0.45	3.022 ± 0.66	61.00	0.002	0.019	−0.62
	c_SyS	0.758 ± 0.14	0.715 ± 0.18	177.00	0.623	0.667	0.10
	w_SyS	0.043 ± 0.03	0.024 ± 0.03	227.50	0.037	0.019	0.40
	m_SyS	0.199 ± 0.14	0.258 ± 0.18	133.50	0.374	0.523	−0.17
	adt_mean	12.952 ± 12.717	9.144 ± 12.05	190.50	0.366	0.523	0.18

Statistical comparisons were performed using the Mann–Whitney U test. Values are reported as median (mean ± SD). δ, Cliff's delta effect size; |δ| ≥ 0.47 indicates a large effect. p-values (p) <0.05 represent statistical significance. mRT_SyS, mean reaction time; c_SyS, percentage of correct responses; w_SyS, percentage of incorrect responses; m_SyS, omission rate (System Monitoring); adt_mean, mean cursor distance from target (Tracking task).

## 3 Results

### 3.1 Exercise duration and energy bar effects

To characterize the impact of energy bar on exercise duration, the duration of all exercise phase and the number of contained peaks was examined (Supplementary Tables S2, S3). In the WO context, 28 out of 80 epochs (35%) were shortened by the energy bar mechanism, with duration ranging from 112 s to 315 s (mean = 195.42 s ± 48.52 s). Across all WO epochs, the average duration was 303.04 s ± 84.03 s. In the SO context, 29 out of 90 epochs (32%) ended early, with shortened duration ranging from 132 s to 355 s (mean = 233.0 s ± 66.9 s).

These results characterize the extent of early termination and the variability of epoch lengths across the dataset. The full distribution of each duration is provided in Supplementary Figure S2 (WO) and Supplementary Figure S3 (SO). The number of epochs falling below the literature-based duration criteria (Shaffer and Ginsberg, 2017; Task Force 1996) for each HRV index are reported in Supplementary Table S8.

### 3.2 Effect of window duration on HRV metric stability

A sensitivity analysis assessed the effect of reducing the analysis window from the full exercise duration to 180 s. The impact was metric-dependent and broadly consistent across WO and SO. Time-domain and non-linear indices showed small typical deviations, with median differences close to zero and relatively limited dispersion, whereas LF and HF exhibited substantially greater variability and wider ranges of differences. LF/HF also showed greater dispersion in SO than in WO. SDNN and SD2 showed small central deviations but some variability across observations, while D2 was comparatively stable. Overall, the 180-s window produced relatively limited changes in most time-domain and non-linear measures, whereas frequency-domain indices were more sensitive to window shortening. These findings support a metric-dependent interpretation of the effect of analysis duration. Detailed results are reported in Supplementary Tables S9, S10.

### 3.3 Validation of task difficulty

Across both operational contexts, exploratory correlations between task difficulty and performance revealed patterns that varied across activation ranges. In the WO condition, associations emerged only for a limited subset of metrics and intervals, suggesting that difficulty-related effects may appear selectively within specific activation states. In the SO condition, a broader set of metrics showed difficulty-related associations across multiple ranges, indicating more widespread patterns under concurrent-demand conditions. Confidence intervals and permutation-based inference provided an estimate of the stability of these exploratory findings, with detailed results reported in Tables 2, 3.

#### 3.3.1 Perceived workload and stress across difficulty ranges

Ratings of perceived workload (BWS) and stress (VAS) are descriptively summarized in Tables 10, 11 by difficulty range and task context. Both measures increased monotonically with task difficulty, supporting the expected progression from Easy to High. When comparing contexts, the addition of secondary tasks in the SO condition resulted in a higher mean perceived stress value in the Medium range. The analysis is purely exploratory and has no statistical significance.

**Table 10:** Mean ± standard deviation of perceived workload (BWS) across difficulty ranges (*Easy, Medium*, and *High*) in the Workload-Only (WO) and Stress-Oriented (SO) conditions.

Difficulty range	WL perceived WO	WL perceived SO
Easy	2.90 ± 1.71	2.69 ± 1.41
Medium	4.54 ± 1.09	5.49 ± 1.27
High	8.72 ± 1.90	8.65 ± 1.43

Values are reported descriptively; no inferential test was applied.

**Table 11:** Mean ± standard deviation of perceived stress (VAS) across difficulty ranges (*Easy, Medium*, and *High*) in the Workload-Only (WO) and Stress-Oriented (SO) conditions.

Difficulty range	WL perceived WO	WL perceived SO
Easy	2.50 ± 2. 10	2.02 ± 1.68
Medium	3.11 ± 2.49	4.62 ± 1.96
High	5.70 ± 2.82	5.75 ± 2.67

Values are reported descriptively; no inferential test was applied.

#### 3.3.2 Validation of task difficulty on performance across activation levels: Workload-Only condition

In the Workload-Only condition, difficulty showed consistent correlations with performance metrics across all activation ranges, differing with varying levels of arousal. In the *Easy* range, difficulty correlated with multiple behavioral metrics, including accuracy-based and tracking metric. A similar but less extensive pattern emerged in the *Medium* range. In the *Hard* range, significant correlations were restricted to the tracking task. Permutation-based inference yielded results consistent with the Spearman correlations; however, none of the effects remained significant after FDR correction, indicating that these findings should be interpreted as exploratory (see Table 2).

#### 3.3.3 Validation of task difficulty on performance across activation levels: stress-oriented condition

In the *SO* context, difficulty showed a broad and consistent impact on performance. In the *Easy* and *Medium* ranges, difficulty was associated with nearly all behavioral metrics, whereas in the *Hard* range the pattern became more selectively. Importantly, several associations in the *Medium* and *Hard* ranges remained statistically reliable after FDR correction. Permutation-based inference yielded results fully consistent with the Spearman correlations, further supporting the robustness of these effects. Full results are reported in Table 3.

### 3.4 Workload-Only condition: HRV-performance correlations

HRV-performance correlations in the WO context varied as a function of both physiological activation range and task type. In the *Easy* range, only LF power showed significant correlations with performance metrics. In the *Medium* range, performance measures were associated with both autonomic indices (SDNN, SD2) and baroreflex-related variability (LF). In the *Hard* range, correlations with autonomic metrics persisted, and an additional association emerged with sample entropy. The significant correlations are all supported by the permutation test (*p*_perm <0.05), indicating greater stability with respect to the distributional assumptions. However, the effects did not overcome the FDR correction (*p*_FDR and *p*_FDR-perm) and have wide 95% CIs; although the lower and upper bounds in the most cases have the same sign, uncertainty remains high, so the associations are reported for exploratory purposes only. Only HRV indices showing at least one significant correlation with performance (*p*_raw <0.05) are included in Table 4; all non-significant indices and metrics were omitted for clarity (for Correlation Matrix see Supplementary Figures S6–S8).

#### 3.4.1 Workload-Only condition: performance differences across activation ranges

Consistent with the analytical framework defined in the Section 2, cross-range comparisons were conducted for all performance metrics. Kruskal–Wallis tests revealed a significant effects of activation range only for omission rate (*m_SyS*). Results are summarized in Table 6.

*Post-hoc* pairwise comparisons indicated that differences in omission rate were driven by the contrast between *Easy* and *Medium* ranges. No significant differences emerged between the *Medium* and *Hard* ranges for any metric, indicating comparable behavioral outcomes at these two ranges. *Post-hoc* results are reported in Table 7.

Descriptive statistics for each activation range are reported in Table 12.

**Table 12:** Descriptive statistics for performance metrics across physiological activation ranges in the Workload-Only condition.

Activation range	mRT_SyS	C_SyS	w_SyS	m_SyS	adt_mean
Easy	2.02 ± 0.43	0.67 ± 0. 21	0.04 ± 0.03	0.27 ± 0.19	17.6 ± 18.6
Medium	2.05 ± 0.62	0.81 ± 0.19	0.04 ± 0.05	0.14 ± 0.16	7.43 ± 12.3
Hard	2.26 ± 0.45	0.76 ± 0.13	0.04 ± 0.03	0.18 ± 0.13	14.46 ± 15.3

Values are reported as mean ± standard deviation.

### 3.5 Stress-oriented condition: HRV-performance associations

HRV-performance correlations in *SO* context varied across physiological activation ranges and performance domains. In the *Easy* range, significant correlations were limited to LF. In the *Medium* range, correlations extended to vagal indices (RMSSD, pNN50, SD1), frequency-domain measures (LF), and non-linear metrics (FuzzyEn and PermEn). In the *Hard* range, significant correlations were mainly observed for vagal and complexity-related indices (RMSSD, HF, and SD1), particularly in relation with System Monitoring accuracy. As in the *WO* context, significant Spearman correlations were supported by permutation tests but did not survive Benjamini–Hochberg correction and are associated with wide confidence intervals, so these associations should also be interpreted as preliminary indications. Table 5 includes only HRV indices showing at least one significant association (*p*_raw <0.05); non-significant metrics were omitted for clarity (for Correlation Matrix see Supplementary Figures S9–S11).

#### 3.5.1 Stress-oriented condition: performance differences across activation ranges

Kruskal–Wallis tests revealed no significant effects of activation range on any performance metric in the Stress-Oriented condition. Effect sizes were small or negligible across all metrics. Results are summarized in Table 8, while the descriptive values are reported in Table 13.

**Table 13:** Descriptive statistics for performance metrics across physiological activation ranges in the Stress-Oriented condition.

Activation range	mRT_SyS	c_SyS	w_SyS	m_SyS	adt_mean
Easy	2.87 ± 0.78	0.75 ± 0.20	0.02 ± 0.02	0.22 ± 0.19	10.45 ± 13
Medium	2.76 ± 0.78	0.76 ± 0.17	0.02 ± 0.03	0.21 ± 0.16	5.98 ± 10
Hard	3.09 ± 0.80	0.69 ± 0.21	0.02 ± 0.03	0.27 ± 0.21	8.21 ± 11

Values are reported as mean ± standard deviation.

### 3.6 Comparison of SDNN-performance associations across SDNN-based and difficulty-based stratification approaches

The SDNN-performance correlations obtained using the SDNN-based GMM stratification were compared with those obtained after stratifying observations by task difficulty (BWS stratification), separately for the WO and SO contexts (Supplementary Tables S11, S12).

In the WO context, the GMM-based stratification showed moderate positive associations that were not replicated by the difficulty-based stratification. Furthermore, the latter showed weaker or opposite correlations for the same metrics. None of these associations remained significant after FDR correction. In SO condition, correlations were generally weak and non-significant for both approaches, with similar patterns in the Medium and Hard/High ranges.

### 3.7 Comparison between Workload-Only and stress-oriented conditions

Comparisons between WO and SO condition were conducted across all three activation ranges. Significant differences were observed for mean reaction times and error rates, with the WO condition showing shorter reaction times and higher error rates. These effects remained significant after applying FDR corrections. Full results are reported in Table 9, and the distributions of behavioral metrics across the three activation ranges in both operational contexts are shown in Figures 5–9. Due to right-skewed distributions, *adt_mean* is reported too using median and interquartile range (IQR) in Table 14.

**Figure 5:**
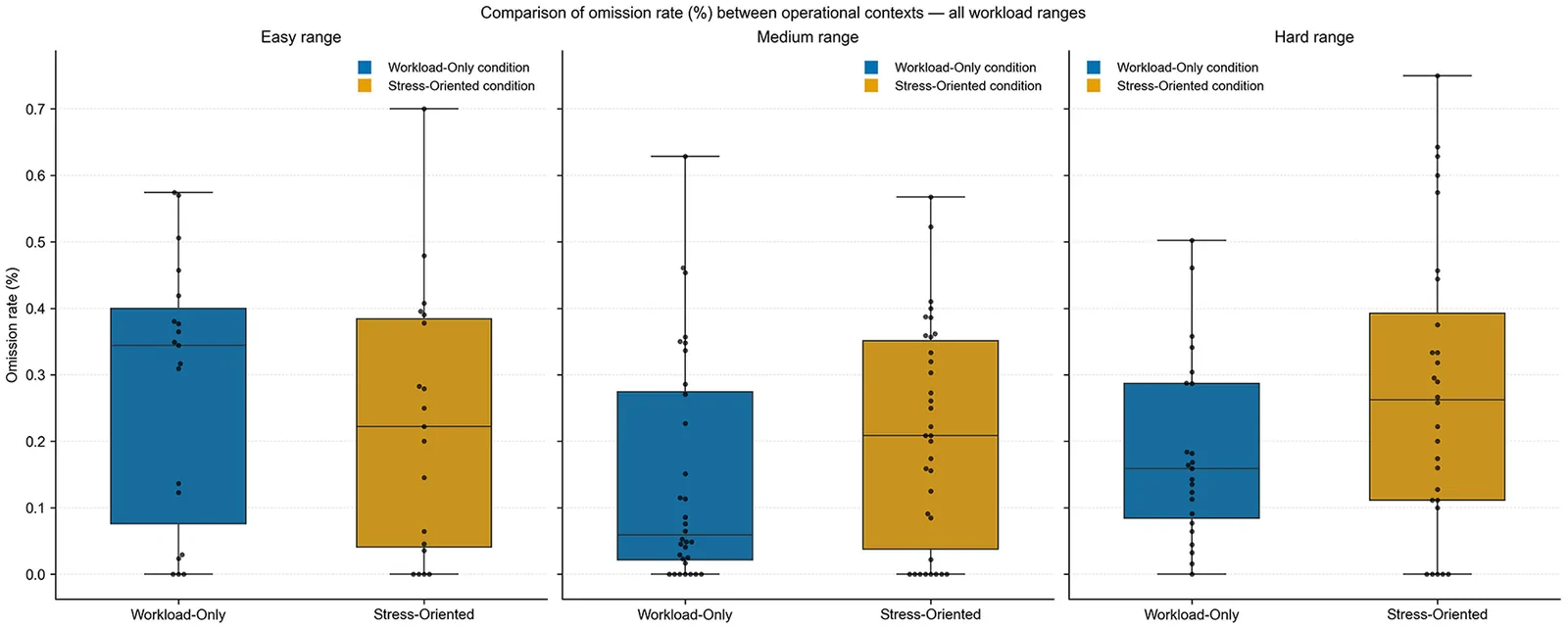
Average cursor distance from target area across the three activation ranges (*Easy, Medium*, and *Hard*) in the Workload-Only (Workload) and Stress-Oriented (Stress) operational contexts.

**Figure 6:**
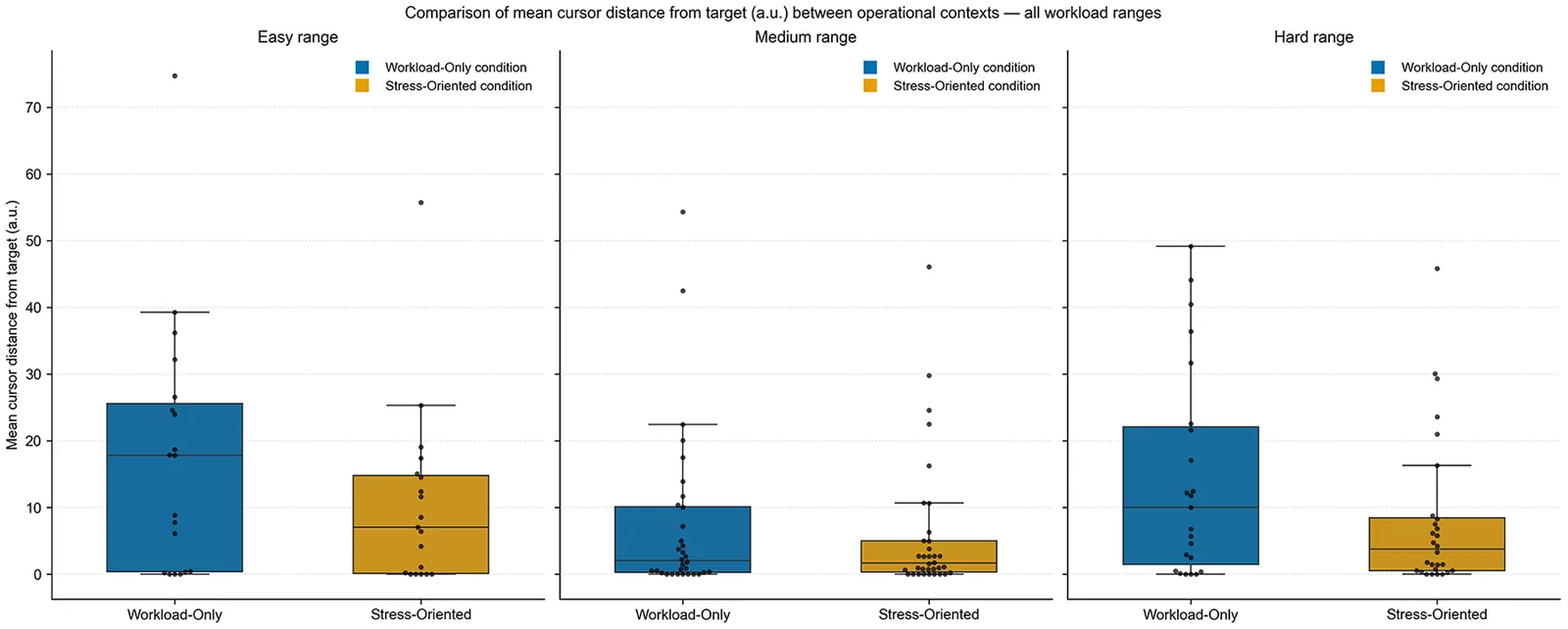
Percentage of correct response across the three activation ranges (*Easy, Medium*, and *Hard*) in the Workload-Only (Workload) and Stress-Oriented (Stress) operational contexts.

**Figure 7:**
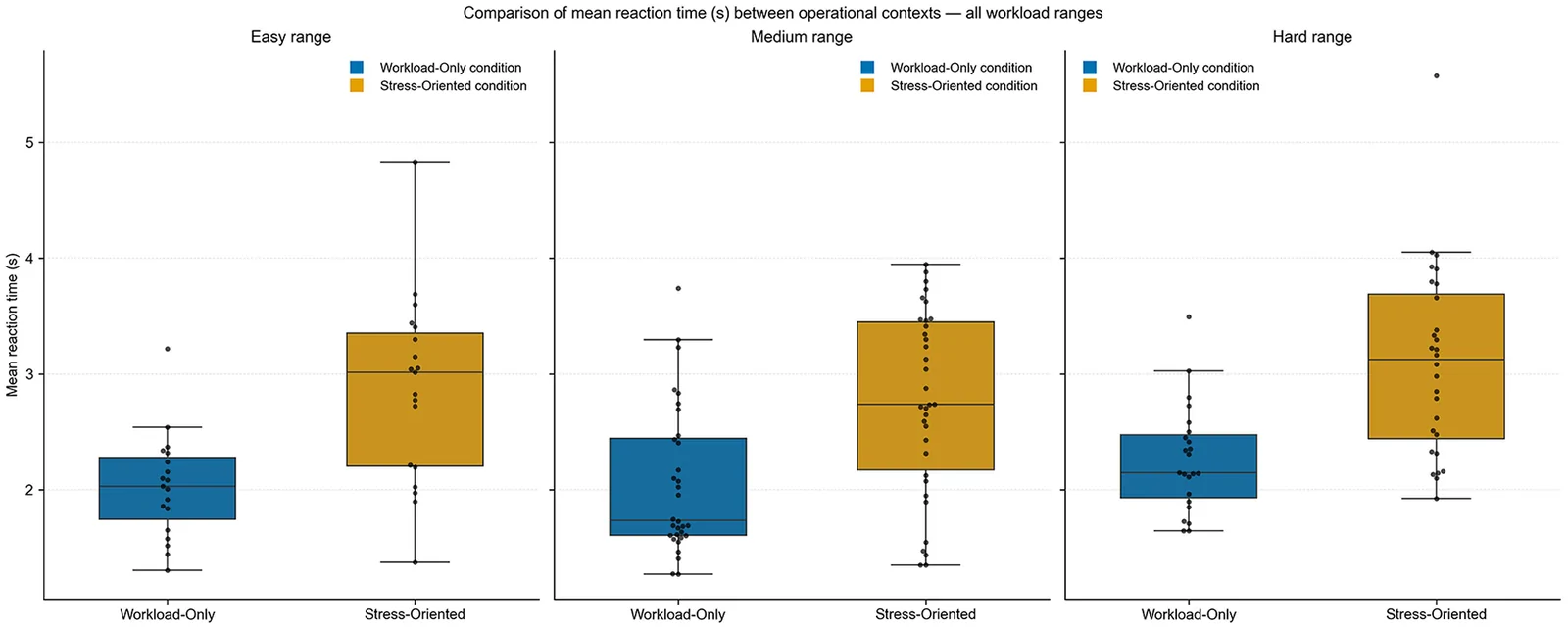
Percentage of missed responses across the three activation ranges (*Easy, Medium*, and *Hard*) in the Workload-Only (Workload) and Stress-Oriented (Stress) operational contexts.

**Figure 8:**
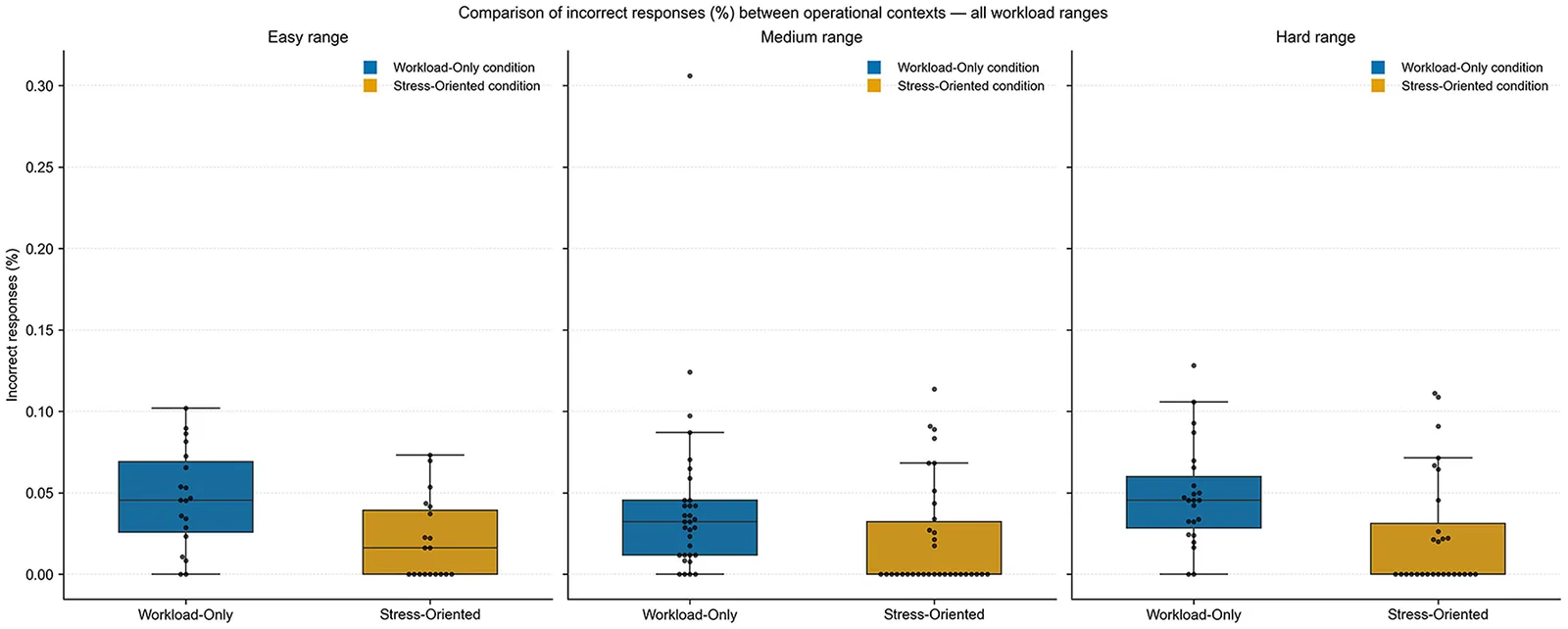
Mean reaction time across the three activation ranges (*Easy, Medium*, and *Hard*) in the Workload-Only (Workload) and Stress-Oriented (Stress) operational contexts.

**Figure 9:**
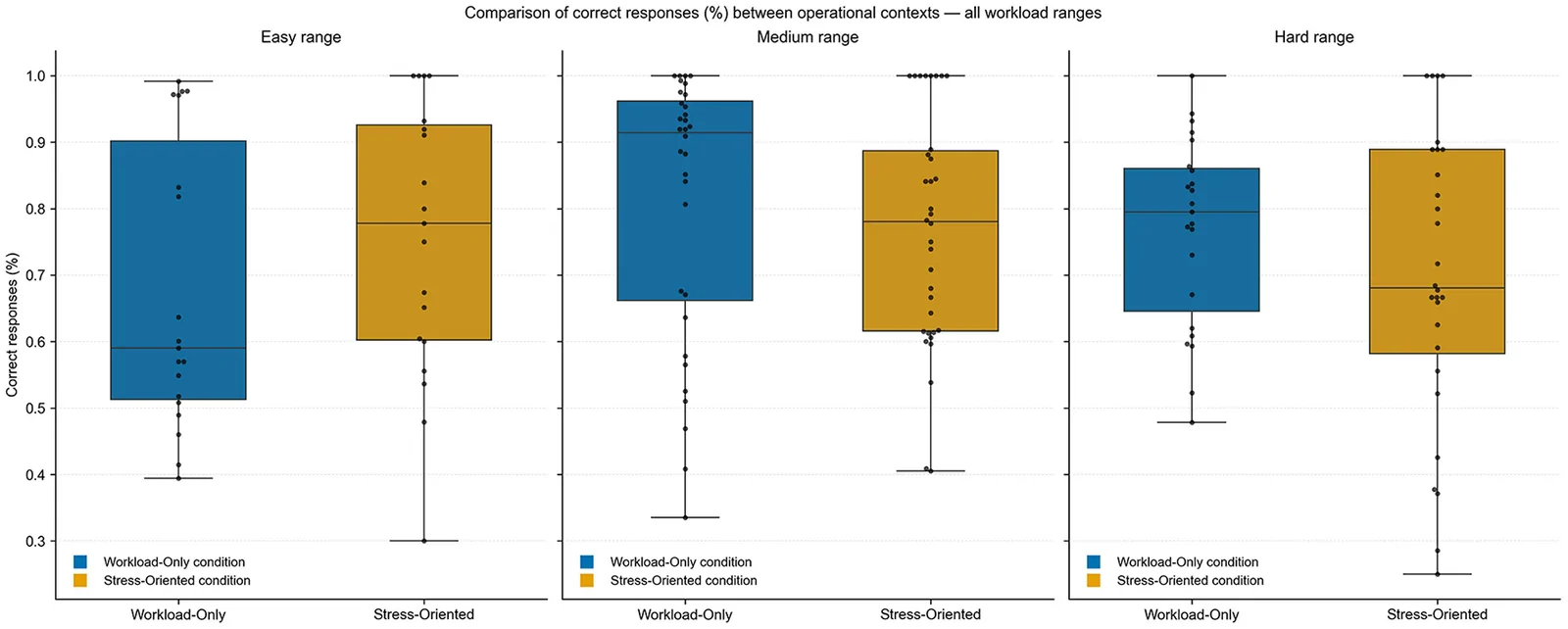
Percentage of wrong responses across the three activation ranges (*Easy, Medium*, and *Hard*) in the Workload-Only (Workload) and Stress-Oriented (Stress) operational contexts.

**Table 14:** Median (IQR) of tracking performance (adt_mean) across activation ranges for WO and SO conditions (reported as median and IQR due to distribution skewness).

Range	Median (IQR) WO	Median (IQR) SO
Easy	18.04 (14.06)	8.79 (14.50)
Medium	3.66 (9.19)	2.34 (5.29)
Hard	8.57 (19.34)	5.09 (11.10)

## 4 Discussion

This study examined how HRV-performance relationships varied across physiological activation ranges, operationalized through SDNN-derived autonomic ranges, across operational contexts differing in terms of task exposure, cognitive interference, and time pressure with the aim of inducing stress. Exploratory analysis of the experimental manipulation reported that perceived workload increased monotonically across difficulty ranges in both conditions, replicating the dose-dependent pattern observed in previous studies (Pontiggia et al., 2024; Di Cesare et al., 2024). Perceived stress also increased level and was descriptively higher in the SO condition in the *Medium* range, as shown by VAS scores. At the behavioral level, task difficulty produced directionally consistent changes in performance metrics in both conditions, though the strength of these associations differed: in the WO condition, correlations were nominally significant but did not survive FDR correction, whereas in the SO condition several associations in the *Medium* range were confirmed after correction—particularly for tracking error (ρ = 0.95; CI 95%: 0.80, 0.98), correct response rate (ρ = −0.76; CI 95%: −0.92, −0.43), and omission rate (ρ = 0.73; CI 95%: 0.38, 0.90)—suggesting that the concurrent presence of stressors amplified the sensitivity of behavioral performance to task difficulty (Warm et al., 2008). Given the small sample size underlying these estimates (*n* = 14–18), correlation coefficients and CI95% values should be treated as indicative rather than precise. These results support the construct validity of AeroStim as a tool for modulating cognitive workload and stress and support the use of difficulty level as a proxy for operational demand in the subsequent analyses.

The definition of the three physiological activation ranges was also subjected to several sensitivity analyses. Although the three-regime structure was specified a priori on theoretical grounds, the GMM provided additional support for this parsimonious solution, with BIC favoring the three-component model. Importantly, classification was highly consistent across alternative SDNN normalization procedures, with 98.4%−100% agreement, while bootstrap analysis indicated relatively stable regime boundaries. These findings suggest that the tripartite classification was not strongly dependent on the specific normalization approach. Nevertheless, because the SDNN normalization and GMM boundaries were derived from the individual recording distributions, the resulting ranges should be interpreted as relative, subject-specific activation states rather than general physiological thresholds.

The duration of the exercise phases and the energy-bar mechanism represent an additional consideration for HRV interpretation. Early termination occurred in approximately one-third of epochs in both conditions, resulting in heterogeneous recording durations. The sensitivity analysis further showed that reducing the analysis window affected HRV metrics in a metric-dependent manner, with frequency-domain indices, particularly LF and HF, showing variability in their deviations than most time-domain and non-linear measures. This is relevant because several shortened epochs did not meet commonly recommended duration criteria for spectral and complexity-based indices. Thus, differences in available recording duration may have contributed to variability in HRV estimates, particularly for frequency-domain measures, and should be considered when interpreting the activation-dependent associations.

Unless otherwise noted, all HRV-performance correlations discussed below are nominal (*p*_raw <0.05), were estimated from small per-range samples (*n* = 14–19), did not survive FDR correction, and carry correspondingly wide 95% CIs; they are therefore treated throughout as exploratory rather than confirmatory, consistent with a possible regime-dependent pattern broadly compatible with neurovisceral integration models (Thayer et al., 2012). In the WO context, HRV-performance correlations changed across activation ranges: LF power showed the only nominal association in the Easy range, potentially reflecting sensitivity to autonomic modulation during lower-demand states (Berntson et al., 1997; Reyes del Paso et al., 2013); associations in the Medium range involved global variability indices (SDNN, SD2) and LF, indicating broader autonomic involvement consistent with compensatory engagement under increasing cognitive demands (Thayer et al., 2012); and Sample Entropy showed an additional nominal correlation in the Hard range, consistent with the possibility that non-linear complexity carries performance-relevant information under sustained cognitive load (Melillo et al., 2011; Zhu and Bo, 2019).

The SO context revealed a more complex autonomic profile. LF showed the only correlation in the Easy range, replicating the WO pattern. In the Medium range, correlations involved vagally mediated indices (RMSSD, pNN50, SD1), SD2, and non-linear complexity measures (Fuzzy and Permutation Entropy), suggesting that parasympathetic and complexity-related indices were more frequently associated with behavioral performance when stressors overlapped with cognitive load, consistent with reports linking stress to vagal withdrawal and reduced complexity (Castaldo et al., 2015; Kim et al., 2018). In the Hard range, correlations were limited to vagal indices (RMSSD, HF, SD1) and LF, relative to error-related metrics, compatible with prior work linking vagal HRV measures to cognitive and behavioral regulation (Lipsitz and Goldberger, 1992; Javorka et al., 2008).

The interpretation of correlations involving SDNN requires additional caution because SDNN was also used to define the activation ranges. The sensitivity analysis indicated that stratification and range restriction may influence SDNN-performance associations within ranges, particularly for the associations observed in the WO condition. These effects highlight the possibility that part of the apparent range-specific relationship may arise from the statistical consequences of conditioning on the same variable used to define the regimes. The association involving *adt_mean* is further susceptible to the influence of extreme values given its right-skewed distribution.

Taken together, these exploratory findings address objectives (i) and (ii) by suggesting that HRV indices associated with performance may vary according physiological activation range and operational context, with implications for adaptive monitoring systems that must account for activation-regime-specific signatures rather than a single HRV index (Teo et al., 2020). At the same time, the sensitivity analyses indicate that both activation-range definition and recording duration can affect the stability and interpretation of these associations, supporting a cautious interpretation of regime-specific HRV-performance patterns.

The relationship between activation level and performance was only partially consistent with the inverted-U hypothesis (Yerkes and Dodson, 1908; Hancock and Ganey, 2003). In the *WO* condition, omission rates were significantly lower in the *Medium* range relative to *Easy* (δ = 0.36), supporting the notion that moderate autonomic activation was associated with better performance; the absence of performance degradation in the *Hard* range is consistent with compensatory effort despite elevated physiological demands (Hockey, 1997). The complete absence of cross-range performance differences in the *SO* condition suggests that higher activation levels there were not associated with proportional behavioral impairment, potentially reflecting motivational and compensatory mechanisms that reduce performance variability across activation states (Matthews et al., 2002). These results only partially address objective (iii): a relative performance advantage was observed at Medium activation in WO, no consistent optimal range emerged across conditions.

Between-condition comparisons revealed a robust speed–accuracy trade-off across all activation ranges: *WO* was associated with shorter reaction times (|δ| = 0.48–0.62, large) and higher error rates (|δ| = 0.41–0.50, medium-to-large), while *SO* showed slower but more accurate responses. This pattern survive to FDR correction and is consistent with associations between stress and risk-averse response strategies (Starcke and Brand, 2012) and impaired response inhibition under concurrent emotional and cognitive demands (Pessoa, 2009). The absence of between-condition differences in omission rate and tracking error suggests that the behavioral differences were selective, affecting mainly response speed and error control rather than the overall execution—consistent with compensatory resource allocation rather than global performance deterioration. Against this background, the qualitatively different patterns of nominal HRV-performance associations observed between conditions—global variability and low-frequency indices in WO, vagally mediated and complexity-based metrics in SO—may point to differences in the physiological processes accompanying performance under different demand structures. Given the exploratory nature of these associations noted above, this interpretation remains hypothesis-generating rather than confirmatory.

These findings are broadly consistent with the heterogeneous HRV literature in aviation. Recent reviews report that different HRV domains can be sensitive to pilot workload, but that results vary substantially across tasks, flight conditions, and participants, with no single metric consistently emerging as the most informative (Wang et al., 2024; Soares et al., 2024). This is consistent with the present finding that performance-related HRV indices differed across activation ranges and between WO and SO, rather than being captured by a single marker. In particular, the involvement of RMSSD, SD1, SDNN, and SD2 in the present analyses is compatible with indices identified as sensitive to workload in real and simulated flight studies (Soares et al., 2024), while the additional contribution of non-linear indices in SO suggests that stress may alter the physiological correlates of performance. Recent real-flight evidence further supports the importance of individual variability: Xu et al. (2026) found above-chance workload and stress classification across pilots, but substantially lower performance under leave-one-subject-out validation than under subject-dependent analysis. Together, these findings support a context- and subject-dependent interpretation of HRV-performance relationships and highlight the need for individualized or adaptive approaches when moving from controlled experiments toward operational monitoring.

### 4.1 Limitations

Several limitations should be considered. First, activation ranges were defined from SDNN, which was also used as a predictor in subsequent analyses. Although the three-regime structure was theoretically specified and showed good stability across normalization methods, this approach introduces potential circularity and range-restriction effects, particularly for SDNN-performance associations. Therefore, independent autonomic markers should be used to validate correlations with SDNN. Second, observations were unevenly distributed across ranges, with a concentration in the Medium range (WO: 43.2%; SO: 42.0%) and small effective sample sizes after aggregation (*n* = 14–19), resulting in wide CIs and limited statistical power. Third, observations were not fully independent: repeated within-subject observations were aggregated by median, but inter-range dependence remain, potentially inflating the apparent consistency of Kruskal–Wallis results. Fourth, exercise duration varied because of early termination by the energy-bar mechanism. Sensitivity analyses showed that this variability may affect HRV estimates, particularly frequency-domain measures, while the absence of respiratory monitoring further limits their physiological interpretation. Finally, the sample comprised only healthy young adults, limiting generalizability; sex was not recorded, precluding assessment of sex-related effects. Overall, these limitations support a cautious interpretation and motivate replication with larger and more diverse samples, controlled recording durations and respiration, and independent markers of autonomic activation.

## 5 Conclusion

This study investigated the relationship between physiological activation, autonomic regulation, and operational performance across two operational contexts—Workload-Only and Stress-Oriented—differing in the nature of the imposed demands. Three principal findings emerge.

First, addressing objective (i), HRV-performance relationships in the WO context varied across physiological activation ranges. LF characterized the *Easy* range, global variability indices (SDNN, SD2) and LF the *Medium* range, and SDNN together with Sample Entropy the *Hard* range. A significant cross-range difference in omission rate, with lower omission at *Medium* than *Easy* activation, provided partial support for a performance advantage at intermediate activation, consistent with the Yerkes-Dodson framework (Hancock and Ganey, 2003). The absence of performance degradation in the *Hard* range is compatible with compensatory effort during elevated physiological demand (Hockey, 1997), suggesting that increased physiological activation may reflect regulatory cost rather than overt performance impairment.

Second, addressing objective (ii), the introduction of additional concurrent demands in the SO condition altered the autonomic correlates of performance without producing systematic differences in performance across activation ranges. In the *Easy* range, LF remained the only autonomic index associated with performance. In the *Medium* range, associations extended to vagal indices, global variability, and non-linear complexity measures, while in the *Hard* range they mainly involved vagal and LF-related indices. The presence of Fuzzy and Permutation Entropy associations in the SO condition, together with preserved behavioral performance, suggests that stress may modify the physiological processes supporting task execution. However, because these HRV-performance associations were predominantly nominal and exploratory, they should be interpreted as hypothesis-generating rather than as evidence of specific autonomic mechanisms.

Third, addressing objective (iii), no single physiological activation range was consistently associated with superior performance across the two operational contexts. Between-condition comparisons instead revealed a robust speed-accuracy trade-off, with WO producing faster but less accurate responses and SO producing slower but more accurate responses. This pattern, together with comparable omission and tracking performance, suggests that the effect of stress was selective rather than indicative of global performance deterioration.

Several methodological findings further qualify these conclusions. The three-regime structure showed good stability across alternative SDNN normalization procedures, and the GMM provided additional support for the parsimonious three-component classification. Nevertheless, because the regimes were derived from subject-specific SDNN distributions, they should be interpreted as relative activation states rather than universal physiological thresholds. Moreover, heterogeneous exercise durations resulting from the energy-bar termination mechanism introduced variability in the available HRV recording windows. The sensitivity analysis indicated that shortening the window to 180 s affected HRV metrics in a metric-dependent manner, with greater deviations generally observed for frequency-domain measures than for most time-domain and non-linear indices. These findings emphasize the importance of standardized recording durations when interpreting spectral HRV measures and regime-specific HRV-performance relationships.

Collectively, the results support the potential utility of an HRV monitoring framework based on physiological activation regimes and incorporating complementary linear and non-linear indices. Rather than identifying a single universal marker of performance, the findings suggest that the physiological correlates of task execution may depend on both activation state and operational context. This distinction may be relevant for adaptive human-machine systems aiming to distinguish behaviorally similar states that differ in their underlying physiological cost.

Future research should extend these findings to larger and more heterogeneous samples, real operational environments, controlled recording durations, and independent measures of autonomic activation, including respiratory monitoring. In particular, future studies should investigate whether activation regimes and HRV-performance relationships generalize across individuals and contexts, and whether stable correlates can be identified using longer and more standardized exercise periods. Developing normalization and thresholding strategies that can be determined independently of dataset-specific distributions will also be important for translating regime-based HRV monitoring into real-time adaptive human-machine systems.

## Data Availability

The datasets presented in this article are not readily available because the request of the approval by the Local Ethical Committee and the Written Informed Consent signed by the participants did not include the possibility of data sharing with third parties. Requests to access the datasets should be directed to arcangelo.merla@unich.it.
