# Supplementary material for: Physiological activation regimes reveal distinct autonomic mechanisms underlying operational performance under cognitive workload and stress
Source: Front Neuroergon. 2026 Oct 2;7:1946611. doi: 10.3389/fnrgo.2026.1946611 (PMC13672659; doi:10.3389/fnrgo.2026.1946611)
Supplement: Supplementary file 1 [file Data_Sheet_1.pdf]

frontiers

Supplementary Material

1 Supplementary Figures and Tables

1.1 Supplementary Figures

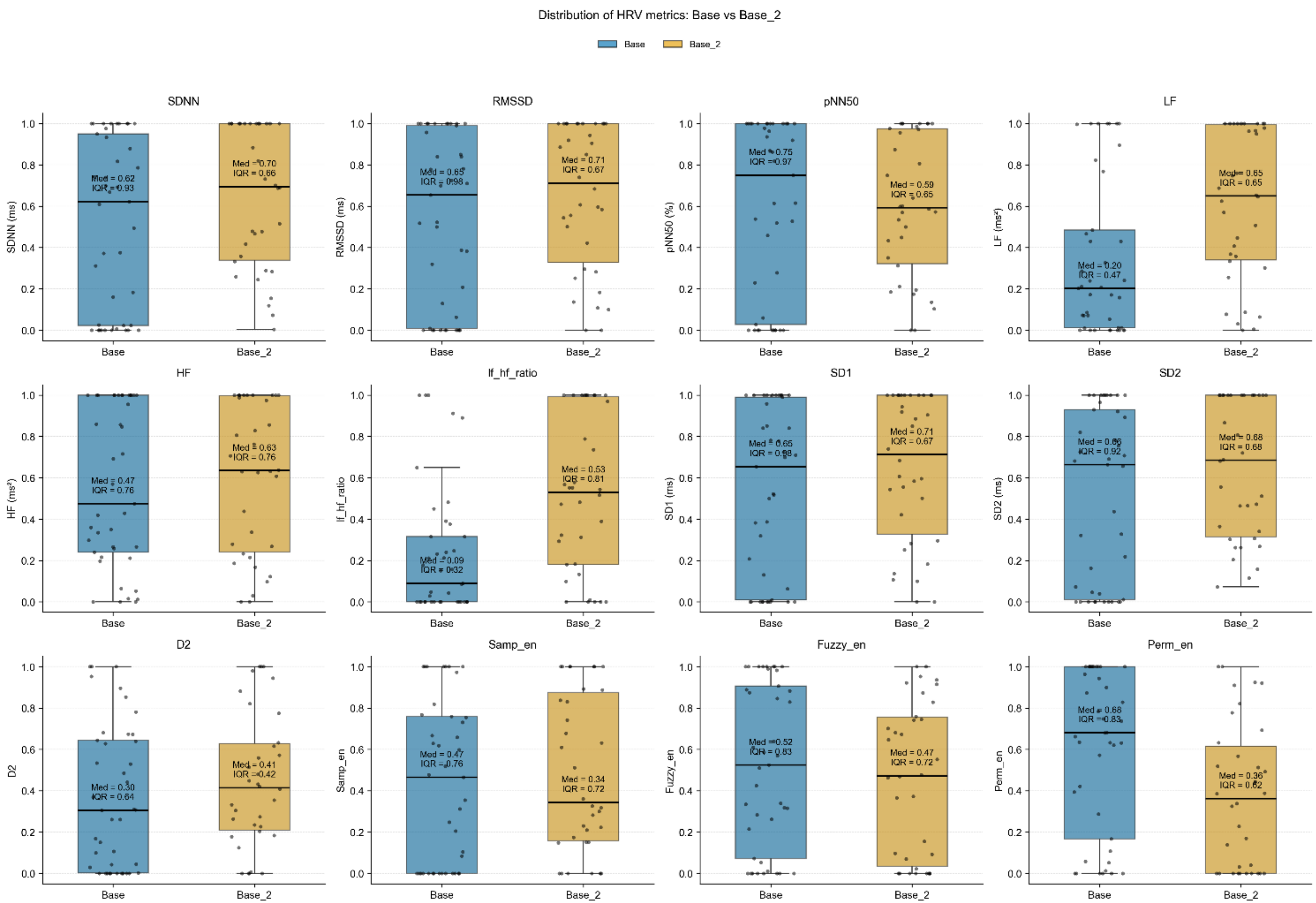

Supplementary Figure S1. Boxplots with overlaid individual observations illustrate the distribution of each HRV metric across participants for the two baseline periods (Base1 and Base2), combining data from both the Workload Only and Stress Oriented contexts. For each metric, the central box represents the interquartile range (IQR), the horizontal line indicates the median, and the whiskers denote variability outside the upper and lower quartiles. Individual data points highlight variability

across participants. Mean values for each condition are reported next to the corresponding box. This figure provides a descriptive comparison of the two baseline periods.

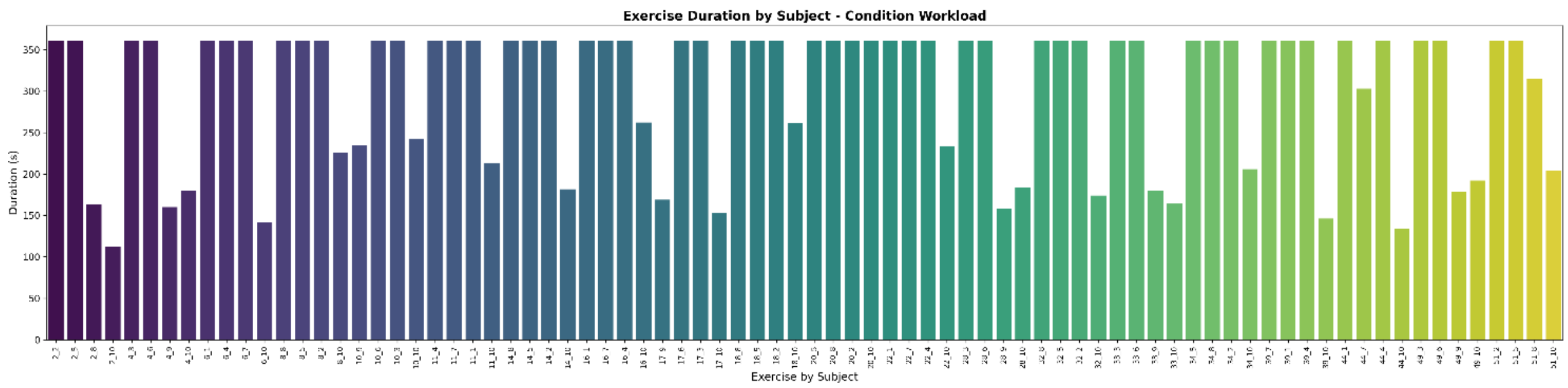

Supplementary Figure S2. Task duration across subjects in WO context. Bar plot showing the duration (in seconds) of each task completed by individual participants during the WO context. On the x-axis, labels represent the unique subject identifier and the difficulty level exercise number (e.g. 2_2 indicate Subject 2, difficulty level 2). Bars reaching values below the standard task duration (360.0s) indicate early task termination (i.e., participant depleted all available energy before completing the full duration). Baseline periods (Difficulty = 0) were excluded from this analysis.

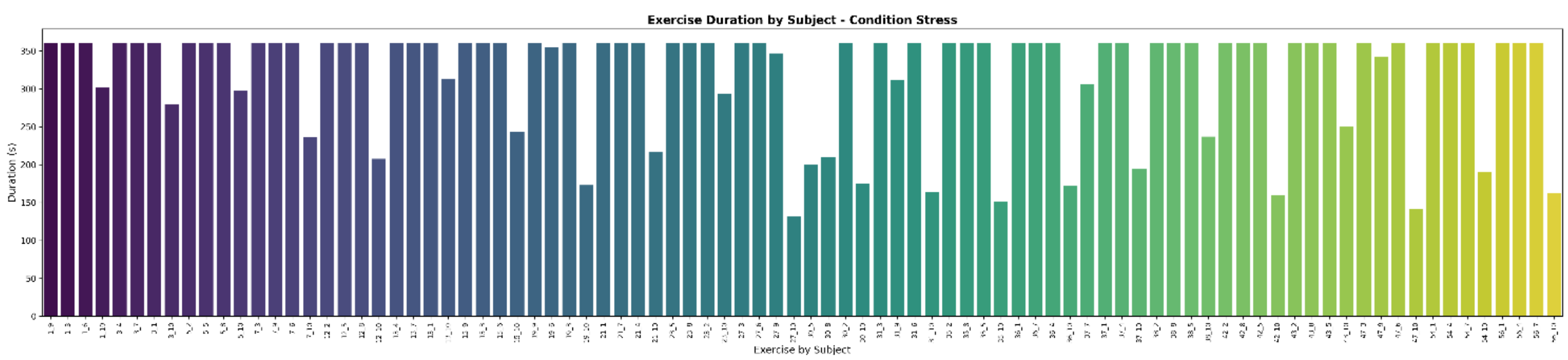

Supplementary Figure S3. Task duration across subjects in SO context. Bar plot showing the duration (in seconds) of each task completed by individual participants during the SO context. On the x-axis, labels represent the unique subject identifier and the difficulty level exercise number (e.g. 1_9 indicate Subject 1, difficulty level 9). Bars reaching values below the standard task duration (360.0s) indicate early task termination (i.e., participant depleted all available energy before completing the full duration). Baseline periods (Difficulty = 0) were excluded from this analysis.

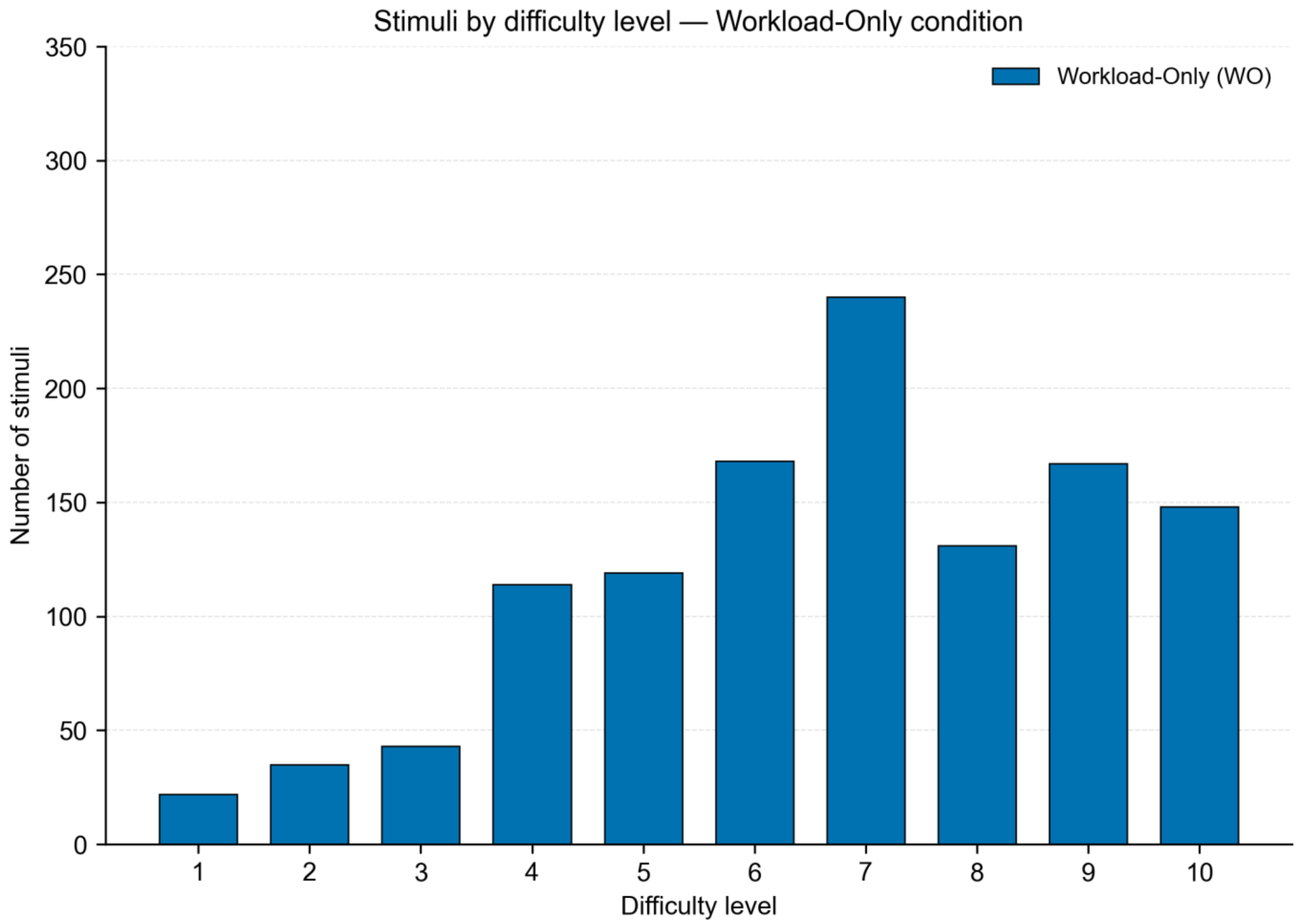

Supplementary Figure S4. Total number of stimuli delivered by the System Monitoring task across the ten exercise difficulty levels in the Workload-Only condition.

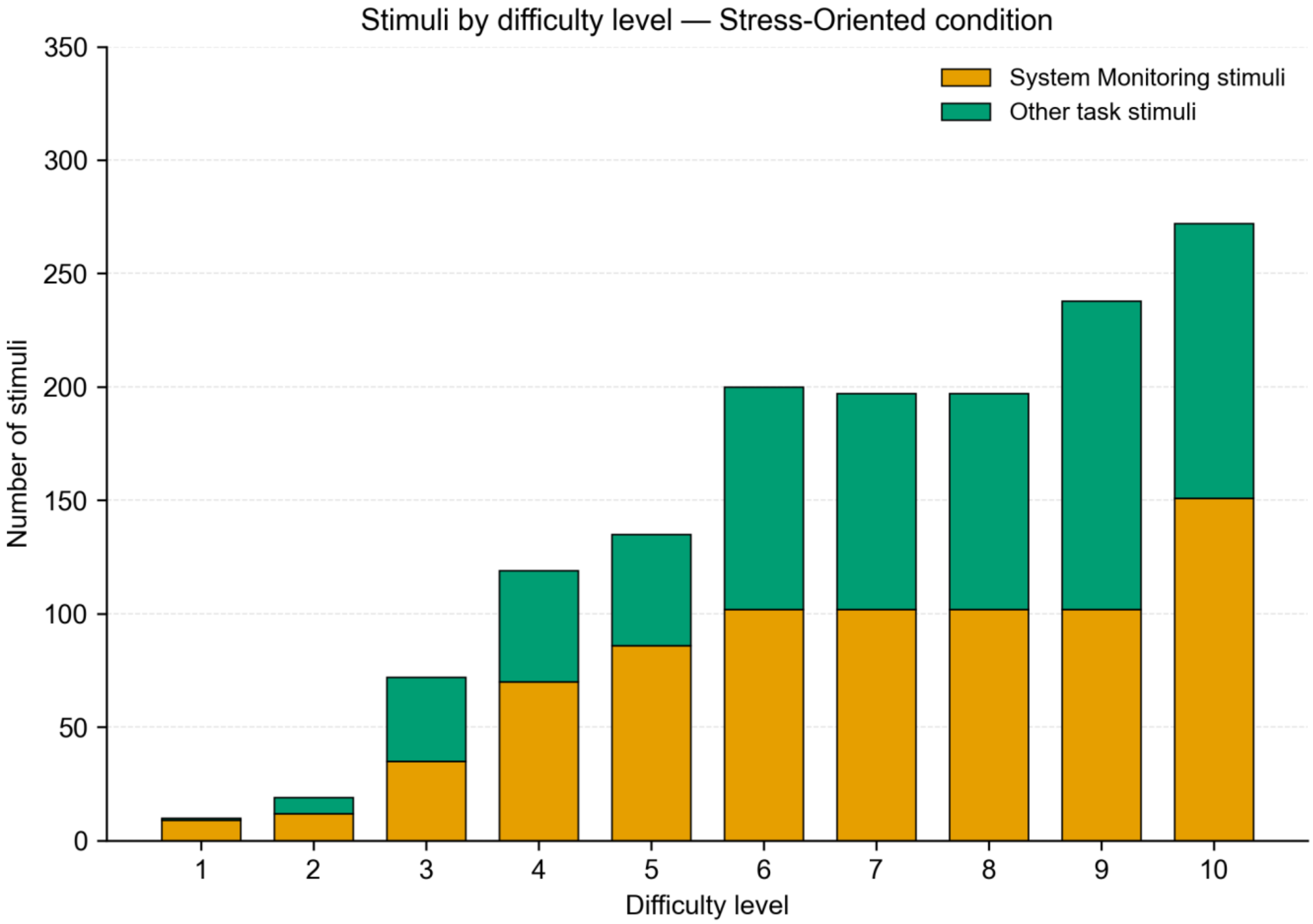
Stimuli by difficulty level — Stress-Oriented condition
System Monitoring stimuli
Other task stimuli
Number of stimuli
0
50
100
150
200
250
300
350
1
2
3
4
5
6
7
8
9
10
Difficulty level

Supplementary Figure S5. Total number of stimuli delivered by the System Monitoring task -salmon- and by the secondary tasks -green- across the ten exercise difficulty levels in the Stress Oriented condition.

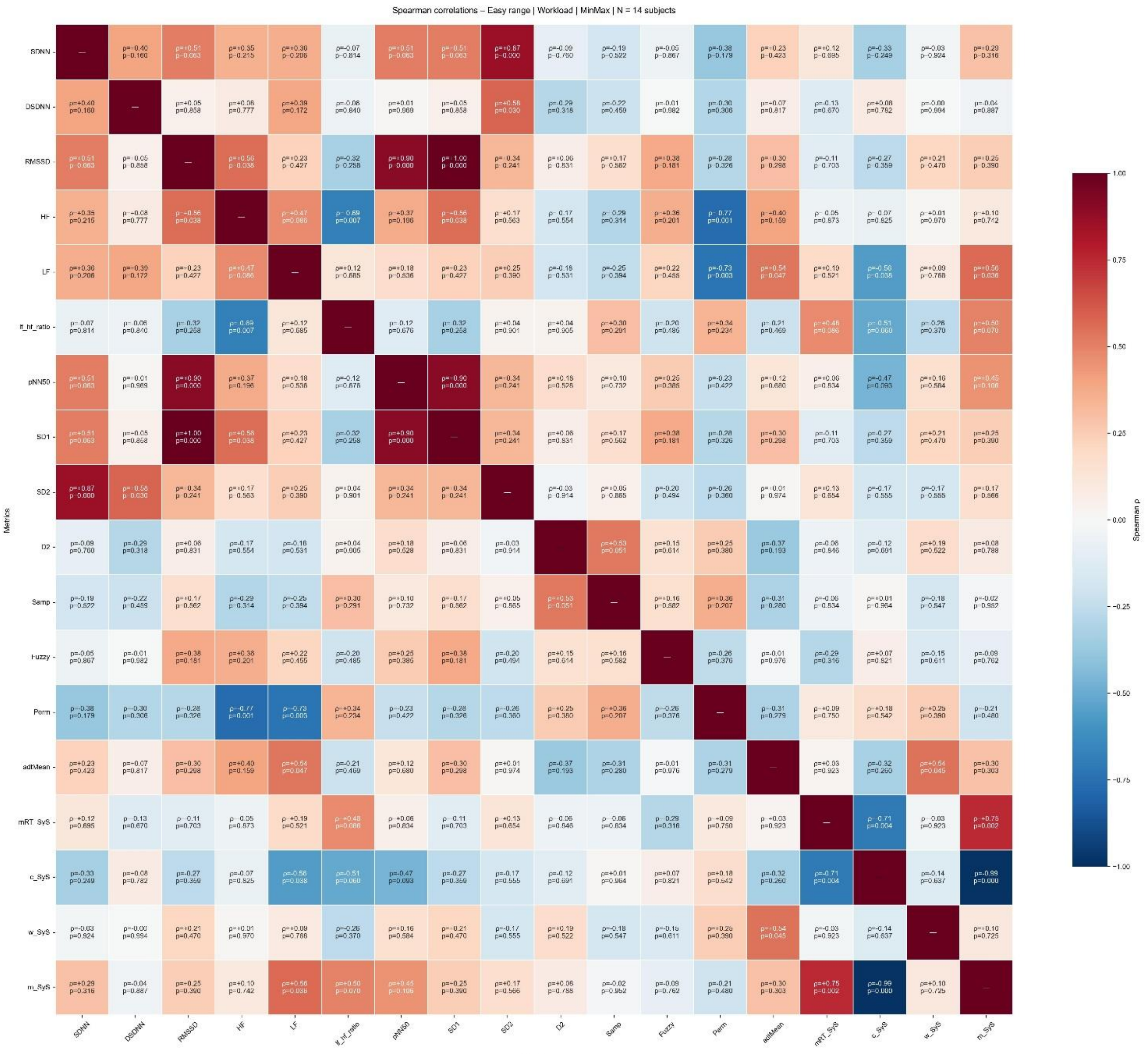

Supplementary Figure S6. HRV-performance correlation matrices in the "Easy" activation interval and WO operating context. Spearman correlation coefficients (ρ) and corresponding p-values (p) are

reported for all HRV indices and behavioral metrics, including non-significant associations. Correlations are calculated on aggregated participant-level data within each activation interval.

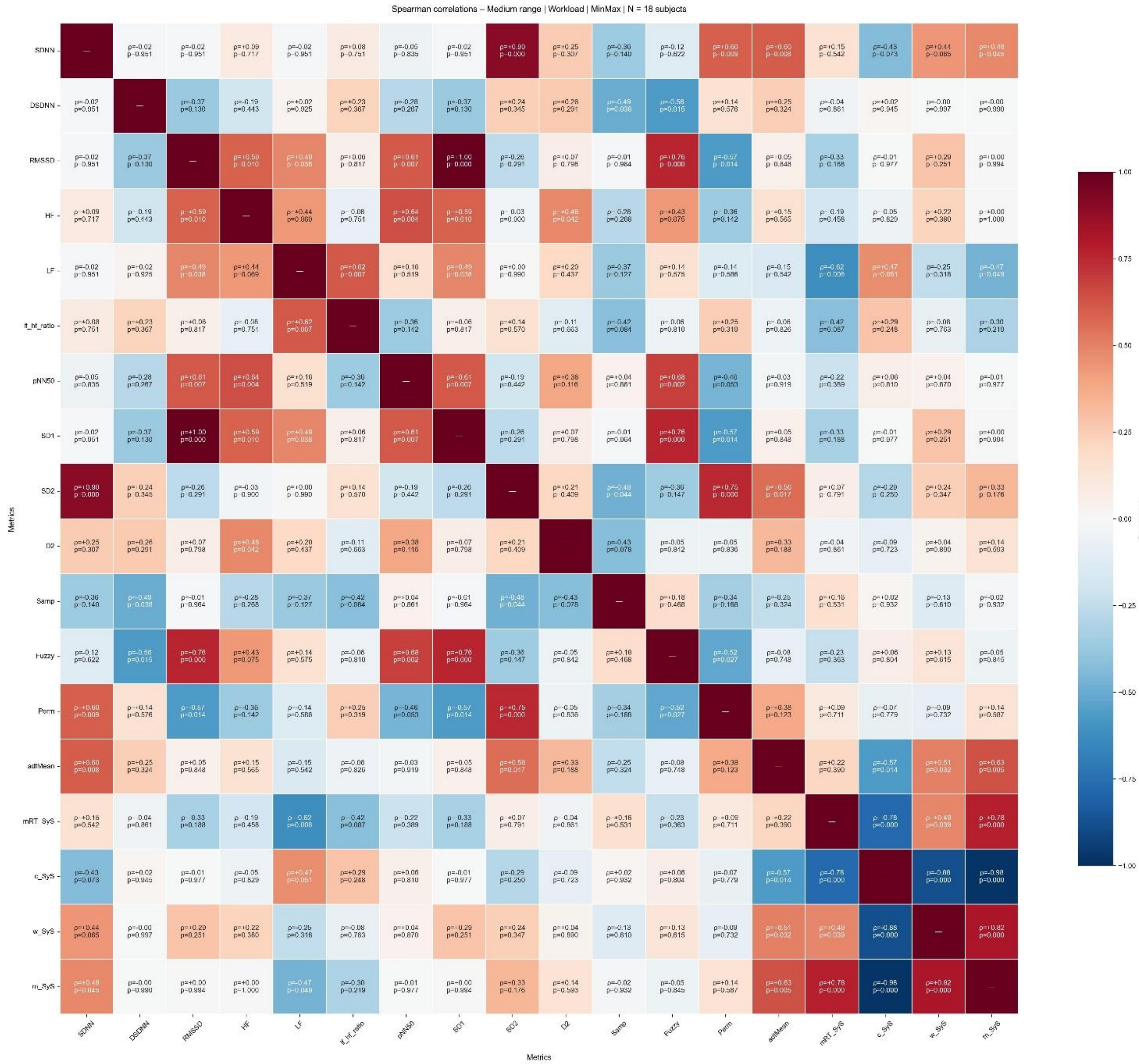

Supplementary Figure S7. HRV-performance correlation matrices in the "Medium" activation interval and WO operating context. Spearman correlation coefficients (ρ) and corresponding p-values

(p) are reported for all HRV indices and behavioral metrics, including non-significant associations. Correlations are calculated on aggregated participant-level data within each activation interval.

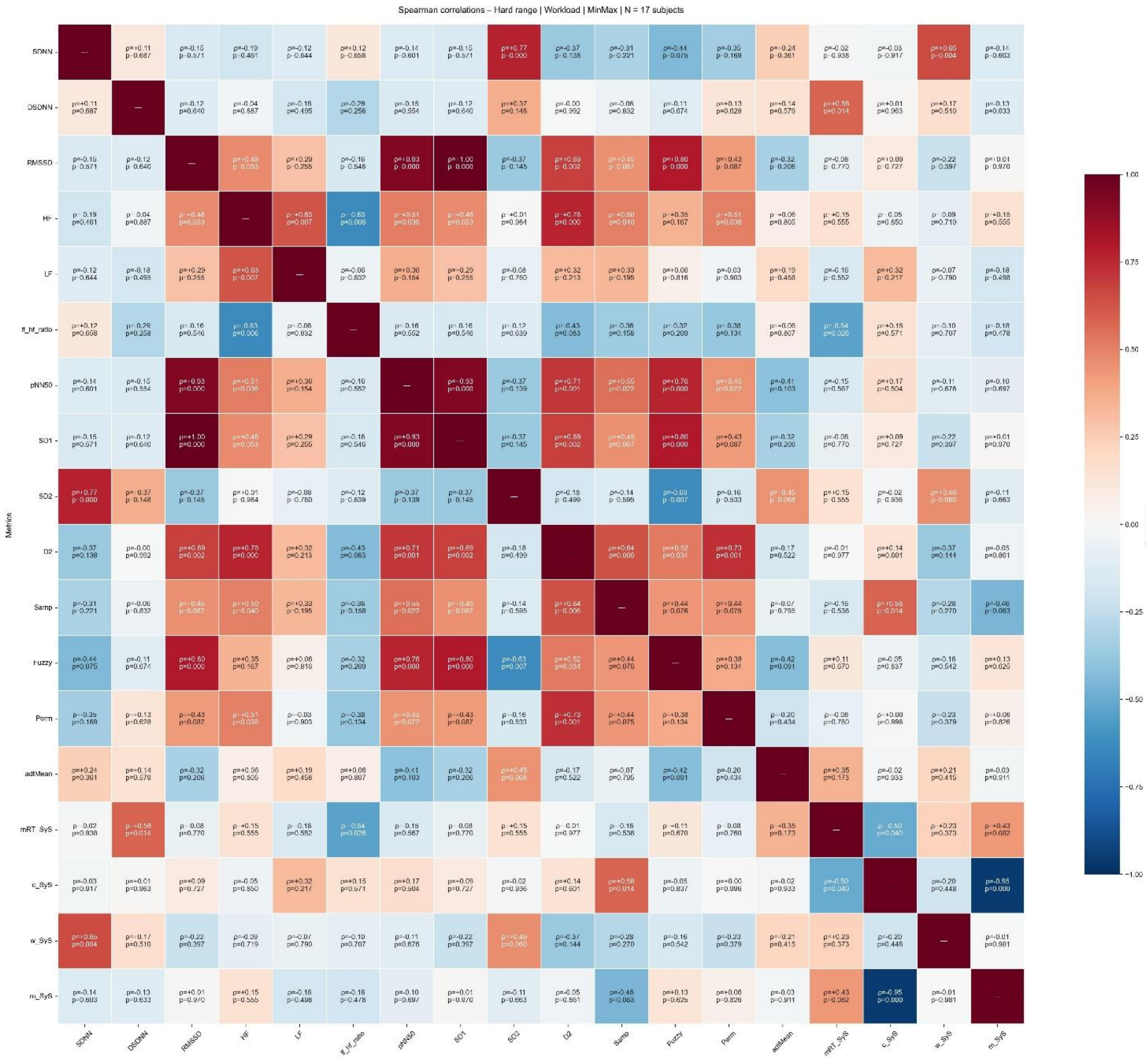

Supplementary Figure S8. HRV-performance correlation matrices in the "Hard" activation interval and WO operating context. Spearman correlation coefficients (ρ) and corresponding p-values (p) are

reported for all HRV indices and behavioral metrics, including non-significant associations. Correlations are calculated on aggregated participant-level data within each activation interval.

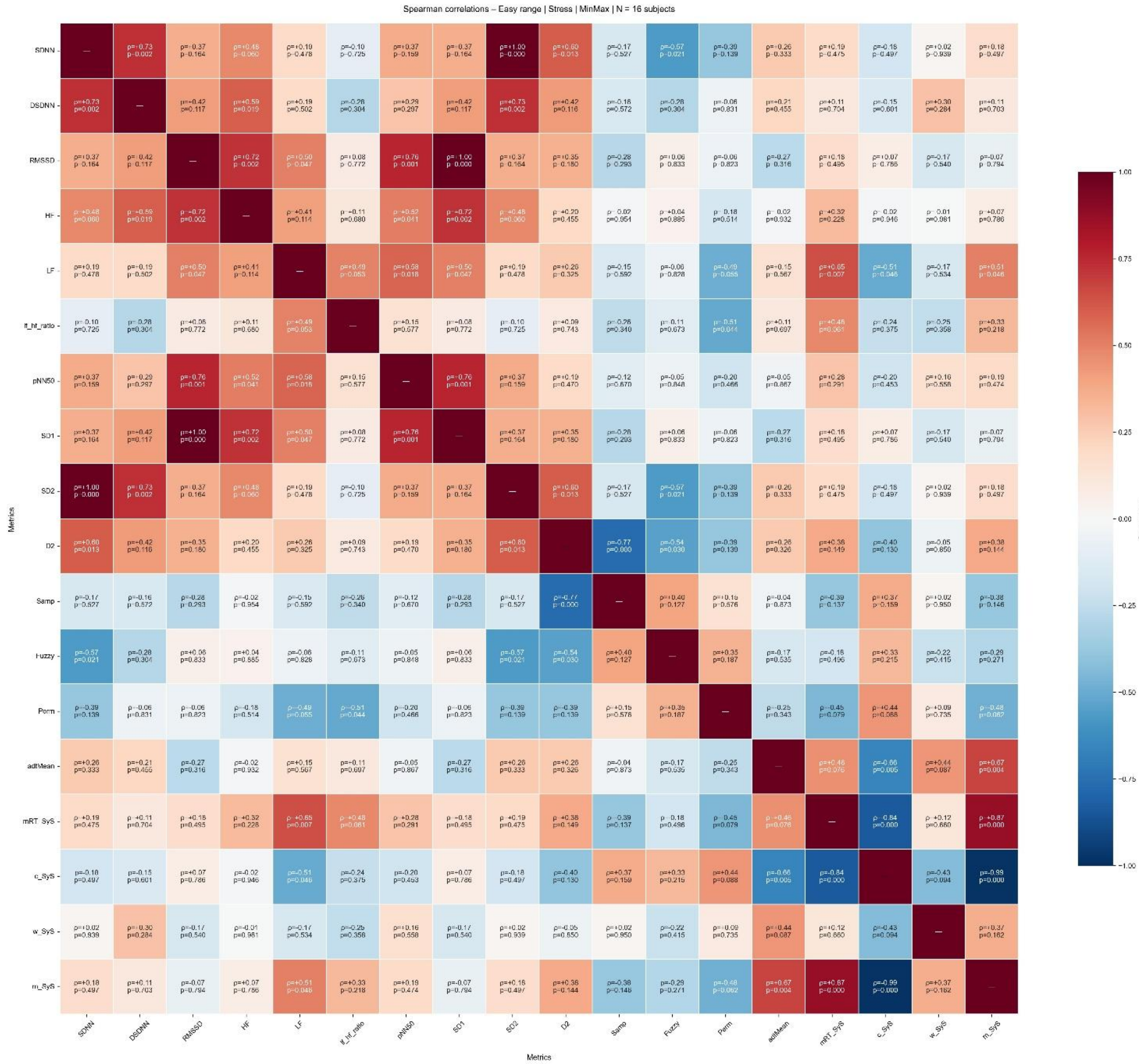

Supplementary Figure S9. HRV-performance correlation matrices in the "Easy" activation interval and SO operating context. Spearman correlation coefficients (ρ) and corresponding p-values (p) are

reported for all HRV indices and behavioral metrics, including non-significant associations. Correlations are calculated on aggregated participant-level data within each activation interval.

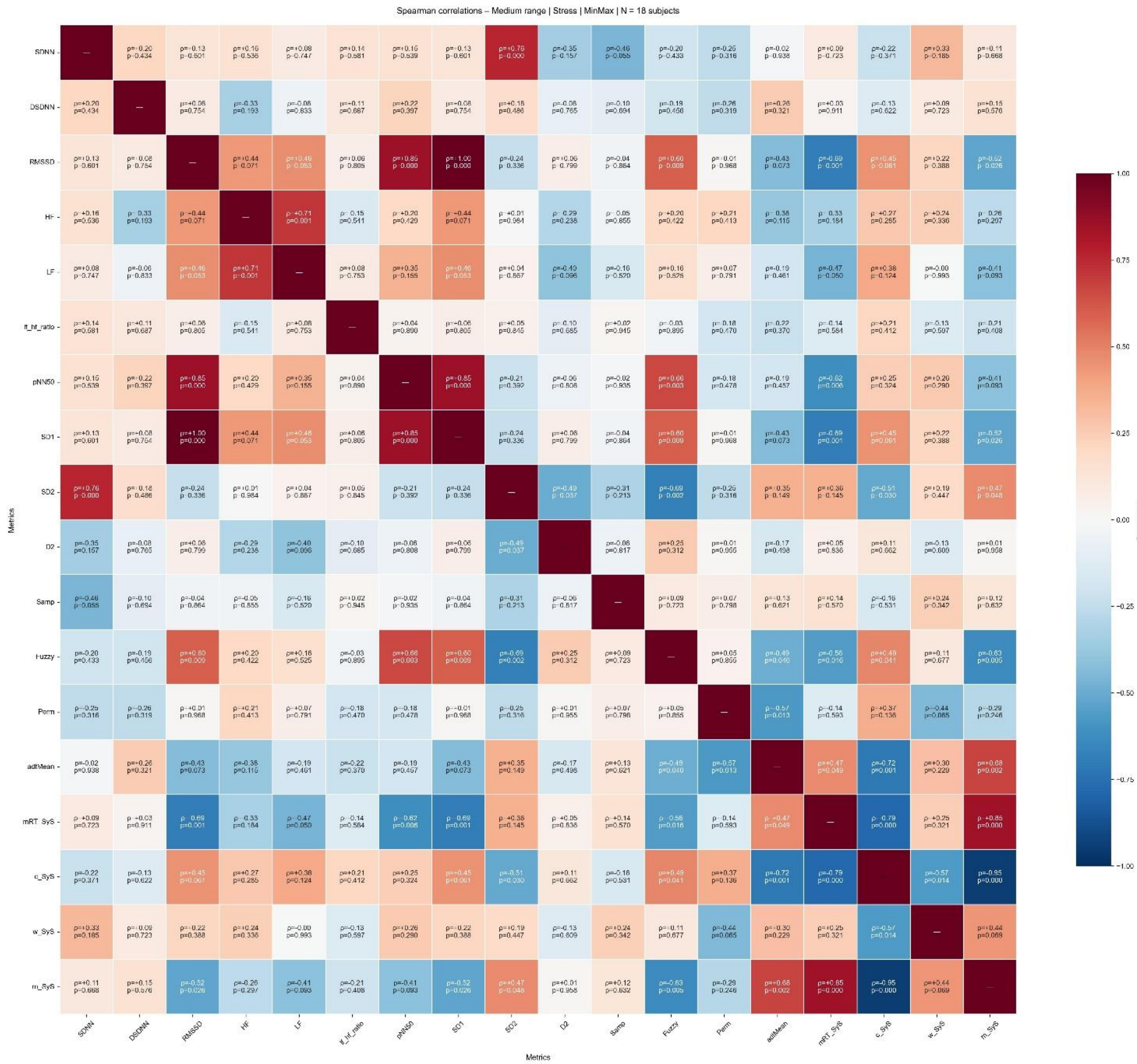

Supplementary Figure S10. HRV-performance correlation matrices in the "Medium" activation interval and SO operating context. Spearman correlation coefficients (ρ) and corresponding p-values

(p) are reported for all HRV indices and behavioral metrics, including non-significant associations. Correlations are calculated on aggregated participant-level data within each activation interval.

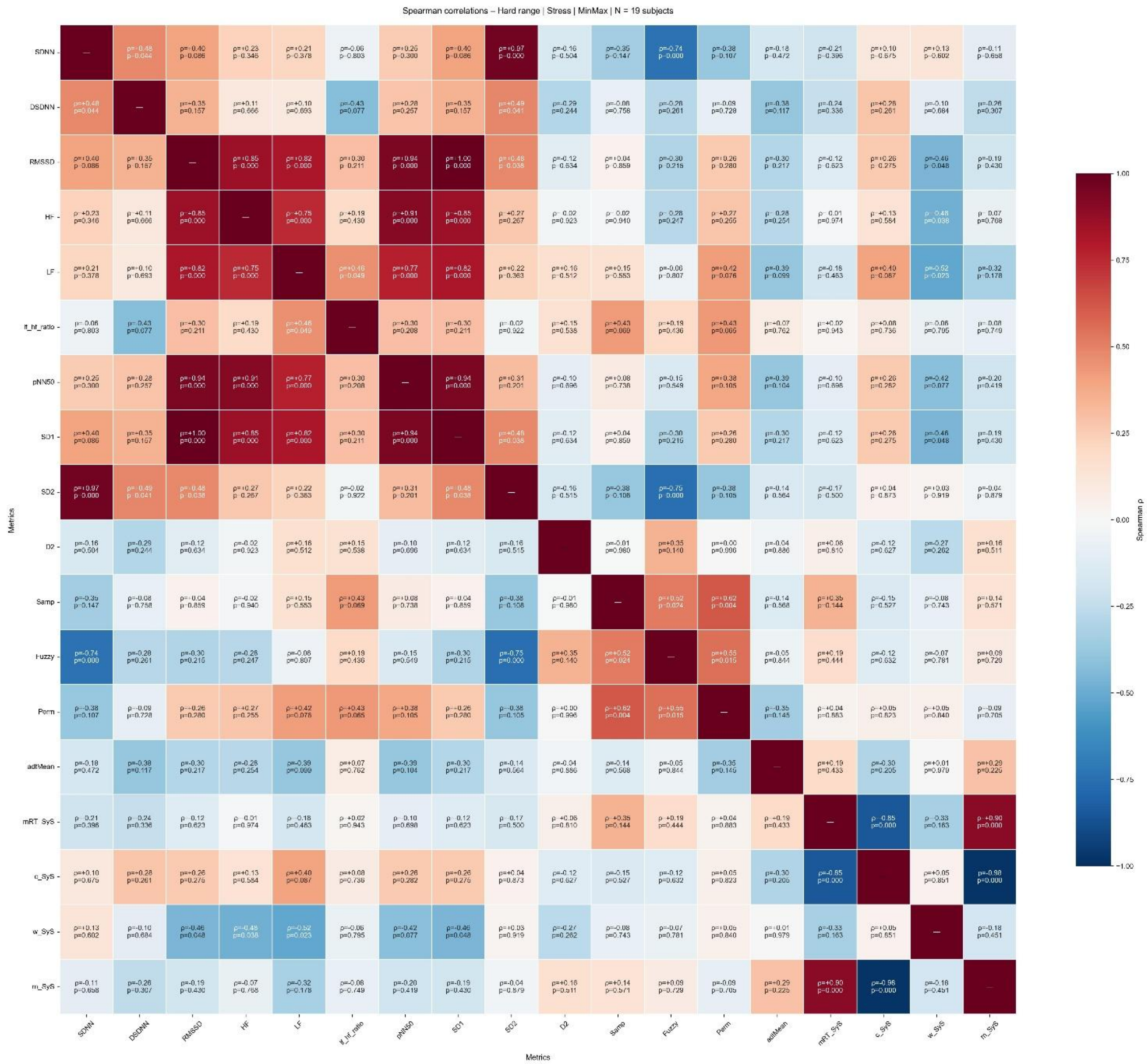

Supplementary Figure S11. HRV-performance correlation matrices in the "Hard" activation interval and SO operating context. Spearman correlation coefficients (ρ) and corresponding p-values (p) are reported for all HRV indices and behavioral metrics, including non-significant associations. Correlations are calculated on aggregated participant-level data within each activation interval.

1.2 Supplementary Table

Supplementary Table S1. Results of the Wilcoxon signed-rank tests assessing whether HRV metrics differed between the two baseline periods. For each HRV metric, the table reports the raw p-value (p_raw) obtained from the Wilcoxon signed-rank test, the p-value corrected by the Benjamini-Hochberg FDR procedure (p_FDR), and the corresponding effect size (rank-biserial correlation). No metric remained statistically significant after the FDR correction ($\alpha = 0.05$). After FDR correction ($\alpha = 0.05$), no metric showed statistically significant differences between baselines.

Metric	p_raw	p_FDR	Effect size	Significant
SDNN	0.073	0.177	-0.351	No
RMSS	0.228	0.342	-0.237	No
pNN50	0.817	0.891	-0.045	No
LF	0.017	0.078	0.469	No
HF	0.488	0.650	0.139	No
LF/HF	0.008	0.078	-0.516	No
SD1	0.228	0.342	-0.237	No
SD2	0.071	0.177	-0.355	No
D2	0.932	0.932	0.018	No
Sample Entropy	0.619	0.743	-0.097	No
Fuzzy Entropy	0.162	0.325	0.277	No
Permutation Entropy	0.019	0.078	0.459	No

Supplementary Table S2. reports for each participant and each exercise in WO context, the operational parameters relevant to the Energy Bar early-termination mechanism. The table includes: (i) the participant identifier, (ii) the exercise number (Exercises 1-4), (iii) the difficulty level assigned to that exercise, (iv) the resulting epoch duration in seconds, (v) the final Energy value at trial termination, and (vi) corresponding number of detected R peaks (R-peaks). The exercise number corresponds to the sequential position within the protocol (Exercise 1, Exercise 2, etc.). This information allows to quantifying which exercises ended before the 6-minute limit, the exact duration of each epoch, the residual energy at termination and count of R peaks.

Subject	N. Exercise	Exercise Difficulty	Duration (s)	Energy	R-peaks
S002	1	2	361.0	999	424.0
	2	5	361.0	960	440.0
	3	8	163.0	0	197.0
	4	10	112.0	0	131.0
S004	1	3	361.0	967	511.0
	2	6	361.0	675	521.0
	3	9	160.0	0	239.0
	4	10	180.0	0	228.0
S006	1	1	361.0	975	457.0
	2	4	361.0	456	475.0
	3	7	361.0	88	486.0
	4	10	142.0	0	243.0
S008	1	8	361.0	305	429.0
	2	5	361.0	965	415.0
	3	2	361.0	1000	424.0
	4	10	226.0	0	259.0
S010	1	9	235.0	0	346.0
	2	6	361.0	882	462.0
	3	3	361.0	995	462.0
	4	10	242.0	0	342.0
S011	1	4	361.0	741	596.0
	2	7	361.0	530	604.0

	3	1	361.0	988	598.0
	4	10	213.0	0	378.0
S014	1	8	361.0	262	376.0
	2	5	361.0	858	359.0
	3	2	361.0	964	357.0
	4	10	181.8	0	191.0
S016	1	1	361.0	991	427.0
	2	7	361.0	636	402.0
	3	4	361.0	818	430.0
	4	10	262.0	0	340.0
S017	1	9	169.0	0	242.0
	2	6	361.0	742	433.0
	3	3	361.0	990	419.0
	4	10	153.0	0	184.0
S018	1	8	361.0	279	548.0
	2	5	361.0	975	541.0
	3	2	361.0	999	572.0
	4	10	261.0	0	400.0
S020	1	5	361.0	976	537.0
	2	8	361.0	571	548.0
	3	2	361.0	988	543.0
	4	10	361.0	120	564.0
S022	1	1	361.0	1000	641.0
	2	7	361.0	550	567.0
	3	4	361.0	737	592.0
	4	10	233.0	0	385.0
S028	1	3	361.0	958	574.0
	2	6	361.0	794	565.0
	3	9	158.0	0	256.0
	4	10	184.0	0	282.0
S032	1	8	361.0	214	546.0
	2	5	361.0	868	528.0
	3	2	361.0	991	518.0
	4	10	174.0	0	265.0
S033	1	3	361.0	984	494.0
	2	6	361.0	897	491.0
	3	9	180.0	0	266.0
	4	10	164.0	0	226.0
S034	1	5	361.0	988	525.0
	2	8	361.0	422	589.0
	3	2	361.0	1000	526.0
	4	10	206.0	0	354.0
S039	1	7	361.0	446	472.0
	2	1	361.0	996	421.0
	3	4	361.0	561	434.0
	4	10	146.0	0	202.0
S044	1	1	361.0	983	528.0

	2	7	303.0	0	453.0
	3	4	361.0	347	505.0
	4	10	134.0	0	188.0
S049	1	3	361.0	899	423.0
	2	6	361.0	523	444.0
	3	9	179.0	0	273.0
	4	10	192.0	0	245.0
S051	1	2	361.0	917	676.0
	2	5	361.0	707	597.0
	3	8	315.0	0	525.0
	4	10	204.0	0	328.0

Supplementary Table S3. reports for each participant and each exercise in SO context, the operational parameters relevant to the Energy Bar early-termination mechanism. The table includes: (i) the participant identifier, (ii) the exercise number (Exercises 1-4), (iii) the difficulty level assigned to that exercise, (iv) the resulting epoch duration in seconds, (v) the final Energy value at trial termination, and (vi) corresponding number of detected R peaks (R-peaks). The exercise number corresponds to the sequential position within the protocol (Exercise 1, Exercise 2, etc.). This information allows to quantifying which exercises ended before the 6-minute limit, the exact duration of each epoch, the residual energy at termination and count of R peaks.

Subject	N. Exercise	Exercise Difficulty	Duration (s)	Energy	R-peak
S001	1	9.0	361.0	467	510.0
	2	3.0	361.0	938	507.0
	3	6.0	361.0	715	523.0
	4	10.0	302.0	0	441.0
S003	1	4.0	361.0	908	495.0
	2	7.0	361.0	810	513.0
	3	1.0	361.0	939	509.0
	4	10.0	280.0	0	419.0
S005	1	2.0	361.0	914	478.0
	2	5.0	361.0	824	502.0
	3	8.0	361.0	658	514.0
	4	10.0	298.0	0	417.0
S007	1	3.0	361.0	917	608.0
	2	9.0	361.0	359	334.0
	3	6.0	361.0	582	580.0
	4	10.0	236.0	0	380.0
S012	1	2.0	361.0	909	581.0
	2	5.0	361.0	643	594.0
	3	8.0	361.0	421	584.0
	4	10.0	207.8	0	343.0
S013	1	4.0	361.0	898	494.0
	2	7.0	361.0	768	513.0
	3	1.0	361.0	939	495.0
	4	10.0	313.0	0	444.0
S015	1	9.0	361.0	332	555.0
	2	3.0	361.0	904	515.0

	3	6.0	361.0	746	524.0
	4	10.0	243.0	0	364.0
S019	1	9.0	361.0	307	705.0
	2	6.0	355.0	0	671.0
	3	3.0	361.0	895	633.0
	4	10.0	173.1	0	313.0
S021	1	1.0	361.0	938	456.0
	2	7.0	361.0	440	361.0
	3	4.0	361.0	881	429.0
	4	10.0	217.0	0	344.0
S023	1	5.0	361.0	764	461.0
	2	8.0	361.0	695	439.0
	3	2.0	361.0	939	428.0
	4	10.0	294.0	0	377.0
S027	1	3.0	361.0	872	397.0
	2	6.0	361.0	21	388.0
	3	9.0	347.1	0	369.0
	4	10.0	132.0	0	141.0
S030	1	5.0	200.1	0	287.0
	2	8.0	210.0	0	281.0
	3	2.0	361.0	831	441.0
	4	10.0	175.0	0	232.0
S031	1	3.0	361.0	884	495.0
	2	9.0	312.0	0	574.0
	3	6.0	361.0	342	498.0
	4	10.0	164.0	0	232.0
S035	1	2.0	361.0	926	529.0
	2	8.0	361.0	442	141.0
	3	5.0	361.0	333	453.0
	4	10.0	151.0	0	122.0
S036	1	1.0	361.0	939	548.0
	2	7.0	361.0	276	529.0
	3	4.0	361.0	829	516.0
	4	10.0	172.0	0	255.0
S037	1	7.0	306.1	0	390.0
	2	1.0	361.0	939	432.0
	3	4.0	361.0	677	463.0
	4	10.0	194.0	0	264.0
S038	1	2.0	361.0	926	383.0
	2	8.0	361.0	654	373.0
	3	5.0	361.0	863	377.0
	4	10.0	237.0	0	247.0
S042	1	2.0	361.0	937	360.0
	2	8.0	361.0	401	373.0
	3	5.0	361.0	541	361.0
	4	10.0	160.0	0	161.0
S043	1	2.0	361.0	939	430.0

	2	8.0	361.0	751	435.0
	3	5.0	361.0	800	435.0
	4	10.0	250.0	0	315.0
S047	1	3.0	361.0	884	593.0
	2	9.0	342.0	0	571.0
	3	6.0	361.0	322	592.0
	4	10.0	141.0	0	242.0
S054	1	1.0	361.0	939	495.0
	2	4.0	361.0	884	560.0
	3	7.0	361.0	691	601.0
	4	10.0	190.1	0	323.0
S056	1	1.0	361.0	939	440.0
	2	4.0	361.0	802	450.0
	3	7.0	361.0	389	443.0
	4	10.0	162.0	0	209.0

Supplementary Table S4. Parameter values used to modulate Tracking-task difficulty across the ten levels ($L_{i,}, i = 1, \ldots, 10$) in the *Workload-Only* condition. Responsiveness reflects the sensitivity of the joystick-cursor control mapping; Random Noise denotes stochastic fluctuations added to the cursor trajectory; Drift Strength models low-frequency directional perturbations producing sustained deviations; and White-Noise Strength quantifies the amplitude of high-frequency stochastic disturbance.

	L1	L2	L3	L4	L5	L6	L7	L8	L9	L10
Responsiveness	8	3	8	8	3	8	10	18	15	24
Random noise	2	2	6	10	4	10	15	25	20	32
Drift strength	0	1	2	5	7	5	15	25	7	40
Withe strength	1	1	2	3	3	2	2	2	10	2

Supplementary Table S5. Parameter values used to modulate Tracking-task difficulty across the ten levels ($L_{i,}, i = 1, \ldots, 10$) in the *Stress-Oriented* condition. Responsiveness reflects the sensitivity of the joystick-cursor control mapping; Random Noise denotes stochastic fluctuations added to the cursor trajectory; Drift Strength models low-frequency directional perturbations producing sustained deviations; and White-Noise Strength quantifies the amplitude of high-frequency stochastic disturbance.

	L1	L2	L3	L4	L5	L6	L7	L8	L9	L10
Responsiveness	2	2	2	4	4	3	3	3	3	10
Random noise	2	2	3	3	3	4	4	4	4	5
Drift strength	4	4	4	4	4	4	4	4	4	4
Withe strength	3	3	3	3	3	3	3	3	3	3

Supplementary Table S6. Quality-control metrics for R-R interval preprocessing in the Workload-Only and Stress-Oriented conditions. For each participant, the table reports - *Total RR*: the total number of detected R-R intervals; *Corrected n (%)*: the number and percentage of correct beats; *Outliers*: the number of outliers beats identified; *Ectopic*: the number of ectopic beats identified; and *% Corrected / Window*: the mean percentage of corrected intervals per window.

Participant	Condition	Total RR	Corrected n (%)	Outliers	Ectopic	% Corrected / Window

S002	Workload	2096	12 (0.6%)	0	12	0.57 ± 0.00
S004	Workload	2451	12 (0.5%)	0	12	0.49 ± 0.00
S006	Workload	2775	6 (0.2%)	1	5	0.22 ± 0.00
S008	Workload	2646	84 (3.2%)	0	84	3.17 ± 0.00
S010	Workload	3550	22 (0.6%)	0	22	0.62 ± 0.00
S011	Workload	3658	13 (0.4%)	2	11	0.36 ± 0.00
S014	Workload	2522	19 (0.8%)	0	19	0.75 ± 0.00
S016	Workload	779	4 (0.5%)	0	4	0.51 ± 0.00
S017	Workload	2392	3 (0.1%)	0	3	0.13 ± 0.00
S018	Workload	3706	27 (0.7%)	0	27	0.73 ± 0.00
S020	Workload	3838	152 (4.0%)	1	151	3.96 ± 0.00
S022	Workload	609	13 (2.1%)	0	13	2.13 ± 0.00
S028	Workload	3607	6 (0.2%)	0	6	0.17 ± 0.00
S032	Workload	3409	7 (0.2%)	1	6	0.21 ± 0.00
S033	Workload	2917	10 (0.3%)	1	9	0.34 ± 0.00
S034	Workload	3557	16 (0.4%)	4	12	0.45 ± 0.00
S039	Workload	2808	8 (0.3%)	0	8	0.28 ± 0.00
S044	Workload	3081	7 (0.2%)	0	7	0.23 ± 0.00
S049	Workload	3356	8 (0.2%)	0	8	0.24 ± 0.00
S051	Workload	3418	11 (0.3%)	0	11	0.32 ± 0.00
S001	Stress	3025	10 (0.3%)	0	10	0.33 ± 0.00
S003	Stress	2927	7 (0.2%)	0	7	0.24 ± 0.00
S005	Stress	2886	5 (0.2%)	0	5	0.17 ± 0.00
S007	Stress	1613	34 (2.1%)	13	21	2.11 ± 0.00
S012	Stress	3393	9 (0.3%)	0	9	0.27 ± 0.00
S013	Stress	3006	54 (1.8%)	1	53	1.80 ± 0.00
S015	Stress	3396	15 (0.4%)	0	15	0.44 ± 0.00
S019	Stress	4294	351 (8.2%)	250	101	8.17 ± 0.00
S021	Stress	936	5 (0.5%)	0	5	0.53 ± 0.00
S023	Stress	2934	13 (0.4%)	0	13	0.44 ± 0.00
S027	Stress	2471	8 (0.3%)	0	8	0.32 ± 0.00
S030	Stress	2696	40 (1.5%)	11	29	1.48 ± 0.00
S031	Stress	3129	13 (0.4%)	1	12	0.42 ± 0.00
S035	Stress	1094	0 (0.0%)	0	0	0.00 ± 0.00
S036	Stress	3249	9 (0.3%)	0	9	0.28 ± 0.00
S037	Stress	2908	47 (1.6%)	1	46	1.62 ± 0.00
S038	Stress	1894	40 (2.1%)	0	40	2.11 ± 0.00
S042	Stress	2153	31 (1.4%)	0	31	1.44 ± 0.00
S043	Stress	2855	8 (0.3%)	0	8	0.28 ± 0.00
S047	Stress	3554	10 (0.3%)	0	10	0.28 ± 0.00
S054	Stress	3387	48 (1.4%)	0	48	1.42 ± 0.00
S056	Stress	2875	8 (0.3%)	0	8	0.28 ± 0.00

Supplementary Table S7. AIC and BIC values from GMMs with 1-6 components. AIC and BIC improve up to three components; BIC reaches its minimum at three components, while additional components offer marginal benefits and greater complexity. This makes the three-componenbt solution parsimonious and consistent with the three-regime theoretical hypothesis.

N components	AIC	BIC
1	181.66	188.23
2	-234.84	-218.42
3	-607.65	-581.39
4	-615.57	-579.45
5	-614.16	-568.20
6	-615.43	-559.62

Supplementary Table S8. Literature-based minimum epoch durations considered for the HRV indices analyzed in the present study. The reported thresholds correspond to ultra-short-term durations shown to provide estimates comparable to conventional 5-min recordings under the validation criteria adopted by Shaffer and Ginsberg (2017), and should therefore not be interpreted as universal minimum durations. For frequency-domain measures, the Task Force of the European Society of Cardiology and the North American Society of Pacing and Electrophysiology recommends approximately 1 min for HF and 2 min for LF, while 5-min recordings are preferred for standardized short-term HRV assessment (Task Force of the European Society of Cardiology, 1996). No validated ultra-short-term threshold was identified for FuzzyEn, PermEn, or correlation dimension (D2); therefore, no specific duration criterion was applied to these indices. The numbers of WO and SO epochs falling below each criterion are reported to characterize the extent to which the available epoch durations met the literature-based requirements for each index.

Index	Literature-based criterion	WO < criterion	SO < criterion
SDNN	60s	0	0
RMSSD	60s	0	0
pNN50	60s	0	0
SD1	90s	0	0
SD2	90s	0	0
LF	90s (UST), 120s (Task Force)	1	0
HF	180s	11	9
LF/HF	180s	11	9
SampEn	180s	11	9
FuzzyEn	Not estabilished	-	-
PermEn	Not established	-	-
D2	No validate UST criterion	-	-

Supplementary Table S9. Sensitivity analysis of HRV metric stability in the Workload Only (WO) condition. For each HRV metric, the table reports the values computed on the full-length exercise window and on a fixed 180-s window, together with summary statistics of their difference (delta): mean, standard deviation, median, median absolute deviation, and minimum/maximum delta.

Metric	Mean whole	Mean 180s	Mean delta	SD delta	Median delta	MAD delta	Min delta	Max delta
SDNN	47,667	46,444	1,224	5,325	0	1,535	-12,966	34,527
RMSSD	31,955	31,738	0,217	1,943	0	0,537	-6,955	6,529
pNN50	13,345	13,391	-0,046	1,839	0	0,307	-7,45	4,42
LF	811,527	786,775	24,752	269,11	0	28,642	-1757,835	730,177
HF	379,415	386,077	-6,662	84,77	0	8,373	-485,006	153,413

lf_hf_ratio	2,827	2,658	0,169	0,676	0	0,078	-2,012	4,725
SD1	22,595	22,441	0,154	1,374	0	0,378	-4,916	4,617
SD2	63,153	61,276	1,877	7,982	0	2,381	-18,752	52,087
D2	0,0009	1,14E-16	0,0009	0,008	0	9,07E-16	-4,48E-15	0,085
Samp_en	1,349	1,379	-0,029	0,153	0	0,036	-0,701	0,23
Fuzzy_en	1,441	1,469	-0,027	0,102	0	0,029	-0,383	0,155
Perm_en	0,955	0,954	0,002	0,007	0	0,004	-0,015	0,021

Supplementary Table S10. Sensitivity analysis of HRV metric stability in the Stress-Oriented (SO) condition. For each HRV metric, the table reports the values computed on the full-length exercise window and on a fixed 180-s window, together with summary statistics of their difference (delta): mean, standard deviation, median, median absolute deviation, and minimum/maximum delta.

Metric	Mean whole	Mean 180s	Mean delta	SD delta	Median delta	MAD delta	Min delta	Max delta
SDNN	48,841	47,272	1,569	6,486	0	1,127	-7,446	48,866
RMSSD	33,95	33,519	0,431	3,552	0	0,704	-3,859	30,85
pNN50	15,784	15,737	0,047	1,675	0	0,725	-4,665	5,821
LF	860,095	807,644	52,451	278,287	0	51,925	-744,781	1752,744
HF	439,397	419,152	20,245	219,717	0	16,94	-594,215	2026,42
lf_hf_ratio	2,498	2,712	-0,214	0,967	0	0,227	-4,206	1,267
SD1	24,005	23,7	0,305	2,511	0	0,494	-2,729	21,814
SD2	64,524	62,23	2,294	9,016	0	1,57	-10,548	65,583
D2	0,0004	7,38E-17	0,0004	0,004	0	9,57E-16	-5E-15	0,0469
Samp_en	1,388	1,413	-0,025	0,172	0	0,034	-0,945	0,398
Fuzzy_en	1,489	1,523	-0,034	0,113	0	0,039	-0,876	0,127
Perm_en	0,955	0,954	0,001	0,008	0	0,003	-0,024	0,029

Table S11. Sensitivity analysis of SDNN-performance associations based on alternative stratification criteria in WO context. ρ = Spearman correlation coefficient between SDNN and performance metrics calculated in SDNN Gaussian Mixture Model (GMM)-based clustering (G) and stratification based on task difficulty (B). p_val = indicates the uncorrected p-value. p_FDR = indicates the p-value after correcting using the FDR.

Range	Metric	ρ_G	p_val_G	P_FDR_G	ρ_B	p_val_B	p_FDR_B
Easy	adt_mean	0.20	0.42	1.0	0.05	0.81	0.97
	mRT_SyS	0.15	0.69	1.0	-0.44	0.05	0.64
	c_SyS	-0.33	0.24	1.0	0.35	0.12	0.72
	w_SyS	-0.02	0.99	1.0	-0.28	0.22	0.79
	m_SyS	0.28	0.31	1.0	-0.36	0.10	0.68
Medium	adt_mean	0.60	<.01	0.51	0.17	0.47	0.91
	mRT_SyS	0.15	0.54	1.0	-0.07	0.77	0.97
	c_SyS	-0.43	0.07	0.71	-0.15	0.53	0.95
	w_SyS	0.44	0.06	0.71	0.02	0.90	0.97
	m_SyS	0.47	0.04	0.71	0.21	0.39	0.91
	adt_mean	0.23	0.36	1.0	0.19	0.42	0.91

Hard (GMM) High (BWS)	mRT_SyS	-0.02	0.93	1.0	0,44	0.06	0.64
	c_SyS	-0.02	0.91	1.0	-0.48	0.04	0.64
	w_SyS	0.65	<.01	0.51	-0.04	0.84	0.97
	m_SyS	-0.13	0.60	1.0	0.45	0.05	0.64

Table S12. Sensitivity analysis of SDNN-performance associations based on alternative stratification criteria in SO context. ρ = Spearman correlation coefficient between SDNN and performance metrics calculated in SDNN Gaussian Mixture Model (GMM)-based clustering (G) and stratification based on task difficulty (B)**.** p_val = indicates the uncorrected p-value. p_FDR = indicates the p-value after correcting using the FDR.

Range	Metric	ρ_G	p_val_G	P_FDR_G	ρ_B	p_val_B	p_FDR_B
Easy	adt_mean	-0.21	0.33	0.78	0.25	0.33	0.81
	mRT_SyS	-0.17	0.44	0.78	0.19	0.47	0.84
	c_SyS	0.10	0.62	0.89	-0.18	0.49	0.84
	w_SyS	-0.22	0.31	0.78	0.02	0.93	0.97
	m_SyS	-0.04	0.84	0.96	0.18	0.49	0.84
Medium	adt_mean	0.44	0.057	0.64	-0.01	0.93	0.97
	mRT_SyS	0.0	1.0	1.0	0.08	0.72	0.90
	c_SyS	-0.19	0.42	0.78	-0.22	0.37	0.84
	w_SyS	0.18	0.45	0.78	0.32	0.18	0.69
	m_SyS	0.20	0.41	0.78	0.10	0.66	0.89
Hard (GMM) High (BWS)	adt_mean	-0.12	0.56	0.87	-0.17	0.47	0.84
	mRT_SyS	-0.32	0.13	0.64	-0.20	0.39	0.84
	c_SyS	0.31	0.15	0.64	0.10	0.67	0.89
	w_SyS	0.06	0.78	0.95	0.12	0.60	0.86
	m_SyS	-0.30	0.16	0.66	-0.10	0.65	0.89
